# Three-dimensional tissue engineered skeletal muscle modelling facioscapulohumeral muscular dystrophy

**DOI:** 10.1093/brain/awae379

**Published:** 2024-11-18

**Authors:** Marnix Franken, Erik van der Wal, Dongxu Zheng, Bianca den Hamer, Patrick J van der Vliet, Richard J L F Lemmers, Anita van den Heuvel, Alexandra L Dorn, Cas G A Duivenvoorden, Stijn L M in ’t Groen, Christian Freund, Bert Eussen, Rabi Tawil, Baziel G M van Engelen, W W M Pim Pijnappel, Silvère M van der Maarel, Jessica C de Greef

**Affiliations:** Department of Human Genetics, Leiden University Medical Center, 2333 ZA, Leiden, The Netherlands; Department of Human Genetics, Leiden University Medical Center, 2333 ZA, Leiden, The Netherlands; Department of Human Genetics, Leiden University Medical Center, 2333 ZA, Leiden, The Netherlands; Department of Human Genetics, Leiden University Medical Center, 2333 ZA, Leiden, The Netherlands; Department of Human Genetics, Leiden University Medical Center, 2333 ZA, Leiden, The Netherlands; Department of Human Genetics, Leiden University Medical Center, 2333 ZA, Leiden, The Netherlands; Department of Human Genetics, Leiden University Medical Center, 2333 ZA, Leiden, The Netherlands; Department of Human Genetics, Leiden University Medical Center, 2333 ZA, Leiden, The Netherlands; Department of Human Genetics, Leiden University Medical Center, 2333 ZA, Leiden, The Netherlands; Department of Clinical Genetics, Erasmus University Medical Center, 3015 GD, Rotterdam, The Netherlands; Department of Pediatrics, Erasmus University Medical Center, 3015 GE Rotterdam, The Netherlands; Center for Lysosomal and Metabolic Diseases, Erasmus University Medical Center, 3015 GE Rotterdam, The Netherlands; Leiden hiPSC Centre, Department of Anatomy and Embryology, Leiden University Medical Center, 2333 ZA, Leiden, The Netherlands; Department of Clinical Genetics, Erasmus University Medical Center, 3015 GD, Rotterdam, The Netherlands; Department of Neurology, University of Rochester Medical Center, Rochester, NY 14642, USA; Department of Neurology, Donders Centre of Neuroscience, Radboud University Medical Centre, 6525 GA, Nijmegen, The Netherlands; Department of Clinical Genetics, Erasmus University Medical Center, 3015 GD, Rotterdam, The Netherlands; Department of Pediatrics, Erasmus University Medical Center, 3015 GE Rotterdam, The Netherlands; Center for Lysosomal and Metabolic Diseases, Erasmus University Medical Center, 3015 GE Rotterdam, The Netherlands; Department of Human Genetics, Leiden University Medical Center, 2333 ZA, Leiden, The Netherlands; Department of Human Genetics, Leiden University Medical Center, 2333 ZA, Leiden, The Netherlands

**Keywords:** three-dimensional tissue engineering, disease modelling, facioscapulohumeral muscular dystrophy, human induced pluripotent stem cells, double homeobox 4, mosaic

## Abstract

Facioscapulohumeral muscular dystrophy (FSHD) is caused by sporadic misexpression of the transcription factor double homeobox 4 (DUX4) in skeletal muscles. So far, monolayer cultures and animal models have been used to study the disease mechanism of FSHD and for development of FSHD therapy, but these models do not fully recapitulate the disease and there is a lack of knowledge on how DUX4 misexpression leads to skeletal muscle dysfunction.

To overcome these barriers, we have developed a 3D tissue engineered skeletal muscle (3D-TESM) model by generating genetically matched myogenic progenitors from human induced pluripotent stem cells of three mosaic FSHD patients.

3D-TESMs derived from genetically affected myogenic progenitors recapitulated pathological features including *DUX4* and DUX4 target gene expression, smaller myofibre diameters and reduced absolute forces upon electrical stimulation. RNA-sequencing data illustrated increased expression of DUX4 target genes in 3D-TESMs compared with 2D myotubes, and cellular differentiation was improved by 3D culture conditions. Treatment of 3D-TESMs with three different small molecules identified in drug development screens in 2D muscle cultures showed no improvements, and sometimes even declines, in contractile force and sarcomere organization. These results suggest that these compounds either have a detrimental effect on the formation of 3D-TESMs, an effect that might have been overlooked or was challenging to detect in 2D cultures and *in vivo* models, and/or that further development of the 3D-TESM model is needed.

In conclusion, we have developed a 3D skeletal muscle model for FSHD that can be used for preclinical research focusing on DUX4 expression and downstream pathways of FSHD in relationship to contractile properties. In the future, we expect that this model can also be used for preclinical drug screening.

## Teaser

Franken *et al.* have developed a 3D tissue engineered muscle bundle model to study facioscapulohumeral muscular dystrophy (FSHD). The model recapitulates key pathological hallmarks of FSHD, including *DUX4* expression and reduced absolute contractile forces, and offers a number of advantages over 2D cultures.

## Introduction

Muscular dystrophies comprise a heterogeneous group of muscle diseases, with close to 70 disease genes identified thus far.^1^ Translational research aiming at development of therapy typically requires studies in (transgenic) animal models, most often mouse models, which at best recapitulate certain key aspects of the disease. From an ethical and economical perspective, it is desirable to use *in vitro* disease models that go beyond the typical monolayer muscle cell cultures, which are limited in complexity and cell culture lifespan. Recently developed 3D tissue engineered skeletal muscle (3D-TESM) models present an attractive opportunity with additional complexity, which might relate more closely to native skeletal muscle than 2D cell cultures.

Facioscapulohumeral muscular dystrophy (FSHD) is such an inherited muscle disease that is challenging to model in monolayer cultures and in mouse models. It is one of the most common muscular dystrophies, with a prevalence of around 1 in 8500 to 1 in 20 000 individuals in Europe.^2,3^ The disease usually arises in the second decade of life, resulting in progressive and often asymmetric muscle weakness, typically starting in the muscles of the face, the shoulder girdles and the upper arms. The disease can progress to lower extremities later in life.^4^ To date, there is no cure available for individuals with FSHD.

Two types of FSHD exist (called FSHD1 and FSHD2); both are caused by genetic and epigenetic changes, converging in the misexpression of *DUX4* in skeletal muscles.^5^ A copy of *DUX4* is situated in every unit of the D4Z4 macrosatellite repeat array on chromosome 4q35.^6-8^ Normally, the D4Z4 repeat array consists of 8–100 units. In FSHD1, the repeat array is contracted to 1–10 units, resulting in an open chromatin structure of the D4Z4 repeat array, leading to DUX4 derepression in skeletal muscles.^8-12^ Approximately 10%–20% of cases are *de novo*, and in ∼50% of *de novo* FSHD1 families a postzygotic contraction of the D4Z4 repeat array is observed.^13,14^ As a result, a *de novo* FSHD1 patient can be mosaic for the D4Z4 repeat array contraction, having both non-affected (non-contracted D4Z4 repeat array) and affected (contracted D4Z4 repeat array) cell populations.^15^

*DUX4* encodes a transcription factor that is normally expressed during early embryogenesis and in the germline, while being silenced in most somatic cells.^16^*In vitro* skeletal muscle cells of FSHD patients display sporadic expression of *DUX4*, which induces multiple toxic cascades of events, including activation of reactive oxygen species, inhibition of myogenesis and RNA nonsense-mediated decay, eventually leading to apoptosis of *in vitro* muscle cells.^17-20^ The exact molecular mechanism by which DUX4 causes muscle pathology and muscle dysfunction in patients and the relative contribution of the many DUX4-regulated pathways to FSHD muscle pathology are still largely unknown.

To study FSHD, various monolayer cell culture models and animal models have been established. These models, however, lack the full capacity of DUX4-mediated muscle pathology in FSHD, limiting the development of therapeutic interventions for this disease. Given that *DUX4* has evolved independently in primates,^6,21^ generation of non-primate animal models that fully recapitulate the disease remains challenging, although the mouse DUX4 homologue, Dux, does seem to have largely overlapping functions.^22,23^ Transgenic mouse models overexpressing *DUX4* exhibit some aspects of FSHD muscle pathophysiology; nevertheless, species differences exist.^24^ Cellular models using primary^25,26^ or immortalized myoblasts^27,28^ and embryonic stem cell-derived skeletal muscle cells^29^ have contributed to our understanding of DUX4 regulation in muscle tissue. The 2D monolayer nature of these cultures, however, brings limitations, including a lack of maturity, limited culturing lifespan, and absence of functional readouts, such as contractile force.

To overcome some of these limitations, *in vitro* 3D skeletal muscle culture methods from human myogenic progenitors have been established, showing aligned, multinucleated myotubes in an extracellular environment.^30,31^ These 3D tissue engineered skeletal muscle (3D-TESMs) models can be cultured for multiple weeks and allow measurement of contractile forces after electrical or chemical stimulation. Studies have already shown relevant contributions of 3D cultures^32,33^ generated from immortalized myoblasts or primary myoblasts for Duchenne muscular dystrophy^32^ and Pompe disease.^33^ However, a 3D-TESM model for FSHD has not yet been reported.

Primary muscle cells have limited proliferation capacity and are difficult to obtain from patients.^34,35^ In this study, we therefore used human induced pluripotent stem cells (hiPSCs), which can differentiate into myogenic cells,^29,36-42^ have an unlimited proliferative capacity^43^ and maintain their pathological phenotype.^29,42,44^ Previously, we showed that we can differentiate hiPSCs into myogenic progenitors (MPs) using a transgene-free and feeder-free protocol, and that these MPs can be expanded and differentiated into 2D myotubes.^45,46^ In addition, we showed that these hiPSC-derived MPs can form highly contractile 3D skeletal muscles with similar specific forces, protein expression and myofibre diameter to 3D skeletal muscles generated from human primary myoblasts.^46^ Of note, a limitation of using hiPSC-derived MPs is that the protocol for obtaining MPs from hiPSCs is time consuming and labour intensive. Additionally, it takes some time to establish and characterize hiPSC lines, and matched affected and non-affected hiPSCs might not be readily available for all genetic backgrounds of FSHD.

**Figure 1:**
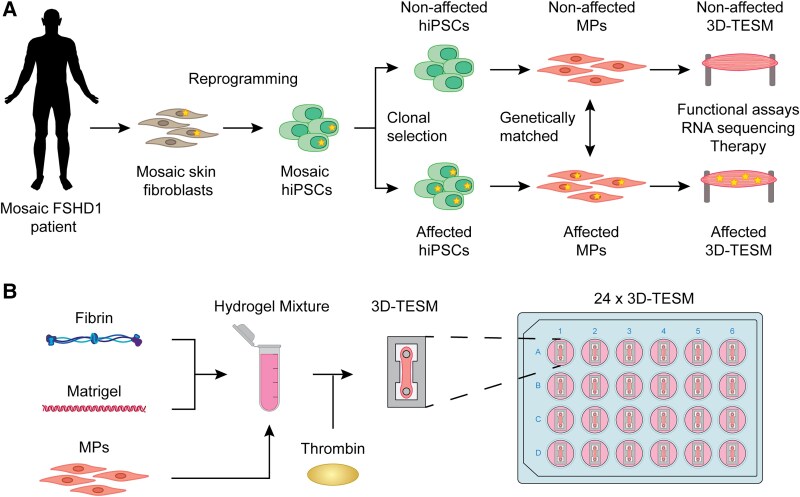
**Schematic overview of the formation of 3D-TESMs.** (**A**) Schematic diagram of the formation of non-affected and affected 3D tissue engineered skeletal muscles (3D-TESMs) from mosaic FSHD1 patients. Skin fibroblasts were obtained from mosaic FSHD1 patients and reprogrammed into human induced pluripotent stem cells (hiPSCs). Single hiPSC colonies were picked, expanded and screened for their D4Z4 repeat array by gel electrophoresis and subsequent Southern blotting, and labelled as either FSHD clone (contracted D4Z4 repeat array) or control clone (normal D4Z4 repeat array). Next, hiPSC clones were differentiated into myogenic progenitors (MPs) using a transgene-free myogenic differentiation protocol. MPs were used to generate functional 3D-TESMs. (**B**) Schematic workflow of the formation of a 3D-TESM in a 24-well plate. MPs were mixed with a hydrogel mixture consisting of fibrinogen and Matrigel. Prior to casting, thrombin was added, and subsequently, the mixture was pipetted into a ‘T-bone’ mould made from polydimethylsiloxane with two flexible pillars, followed by differentiation to a functional 3D-TESM. FSHD = facioscapulohumeral muscular dystrophy type 1.

Here, we describe a 3D-TESM model using hiPSC-derived MPs produced from three independent mosaic FSHD1 patients (Fig. 1). These 3D-TESMs were used to compare the development and strength in relationship to DUX4 expression in affected compared with non-affected MPs. 3D-TESMs grown from non-affected and affected MPs showed similar relative sizes over time, myogenic differentiation marker expression and titin staining. In contrast, affected 3D-TESMs revealed DUX4-related disease pathology, including absolute contractile force reduction in most affected 3D-TESMs, smaller fibre sizes and reduced sarcomere length. Finally, we treated 3D-TESMs with small molecules known to decrease DUX4 and DUX4 target gene expression levels in 2D culture conditions. However, we observed a general negative effect on muscle functionality in both affected and non-affected 3D-TESMs, showing that these compounds potentially have negative impacts on the formation of 3D-TESMs that might have gone unnoticed or were difficult to detect in 2D cultures and *in vivo* models. Alternatively, further refinement of the 3D-TESM model might be required for effective preclinical drug screening.

## Materials and methods

### Ethical statement

Fibroblasts were isolated from human skin biopsies donated by three anonymous mosaic FSHD1 patients. Two of the three fibroblast lines used in this study were provided by the Fields Center for FSHD research biorepository and previously described^44^ (ethical approval RSB00059324, Research Subjects Review Board, University of Rochester). The third fibroblast line was provided by the Dutch Center of Expertise for FSHD (ethical approval by Medical Review Ethics Committee region Arnhem–Nijmegen). Information on the patients is listed in Supplementary Table 1.

### Formation of 3D tissue engineered skeletal muscles

Information on the generation of hiPSCs, the differentiation of hiPSCs to MPs and the culturing and differentiation of MPs can be found in the Supplementary material. Information on the naming of hiPSCs is listed in Supplementary Table 2.

The direct peeling method was used for generation of polydimethylsiloxane (PDMS; Dow Corning) moulds as previously described.^47^ Briefly, T-bone-shaped chambers on a circular-based negative mould of acrylonitrile butadiene styrene (ABS; Ultimaker) were 3D printed using an Ultimaker 2^+^ FDM printer (Ultimaker) equipped with a 0.250 mm nozzle. Uncured PDMS was prepared by mixing curing agent with the prepolymer in a ratio of 1:10 w/w according to the manufacturer’s instructions, degassed, and poured directly over ABS-printed moulds. A second degassing step was performed to remove final air bubbles, after which uncured PDMS was cured at 75°C in an oven for 2 h. After curing, PDMS moulds were carefully peeled off from the ABS negative mould. Single chambers (with a volumetric capacity of 50 µl, containing two cylindrical pillars with a diameter of 1 mm and a height of 3.2 mm) were cut out and fixed inside a 24-well plate (CELLSTAR; Greiner Bio-One), using uncured PDMS as glue, and allowed to attach for 24 h at room temperature. PDMS moulds were sterilized before use in cell culture by rinsing in 70% ethanol for 15 min, washing with PBS, and treating with UV for 15 min, after which PDMS moulds were incubated in 1% Pluronic F-127 (Sigma-Aldrich) for ≥1 h at room temperature.

For generation of 3D-TESMs, a hydrogel mixture consisting of bovine fibrinogen (Sigma-Aldrich) dissolved in Dulbecco’s modified Eagle’s medium (DMEM) high glucose (final concentration 2 mg/ml), Matrigel growth factor reduced (20% v/v; Corning Life Sciences), thrombin from human plasma (Sigma-Aldrich) dissolved in 0.1% bovine serum albumin in PBS (1% v/v, 0.5 U/ml final concentration) and MP proliferation medium (69% v/v) was used. All hydrogel solutions and materials, including tubes and micropipette tips, were incubated and/or defrosted on ice for 30 min and were kept on ice for the duration of the experiments. MPs were detached using TrypLE reagent (1:1 diluted in PBS) (Life Technologies) and suspended in MP proliferation medium (600 000 cells/mould). Next, MPs were initially mixed with fibrinogen and Matrigel, after which thrombin was added. The cell–hydrogel mixture was pipetted directly into the PDMS chamber and incubated at 37°C with 5% CO_2_ for 30 min to polymerize, after which MP proliferation medium supplemented with 6-aminocaproic acid (1.5 mg/ml final concentration; Sigma-Aldrich) was added. After 48 h, MP proliferation medium was replaced with 3D differentiation medium [DMEM high glucose supplemented with 1% ITS-X (Gibco), 1% knockout serum replacement (Gibco), 1% penicillin-G (Sigma-Aldrich), 6-aminocarproic acid (2 mg/ml; Sigma-Aldrich) and SB431542 (10 µM; Selleckchem)]. 3D-TESMs were incubated on agitation at 65 rpm (Celltron, orbital shaker, infors HT) at 37°C with 5% CO_2_, and half of the medium volume was refreshed at Days 3, 5, 7, 10 and 12 of differentiation.

### Electrical stimulation and force measurements of 3D tissue engineered skeletal muscles

After 14 days of differentiation, 3D-TESMs were subjected to a single pulse (frequency of 1 Hz) followed by a tetanic pulse (frequency of 20 Hz) of electrical stimulation using an Arduino Uno Rev3 equipped with an Adafruit motorshield V2 with supplied software (both from Adafruit). The displacement of one of the pillars was captured at 60 frames/s using Thorlabs Microscope filer cubes (Thorlabs) attached to a microscope with assisted Thorlabs software (Thorlabs). Displacements were analysed either by ImageJ Fiji software^48^ or with a modified Python script.^49^ Directly after stimulation, the position of each 3D-TESM at the pillar was imaged at ×4 magnification using the EVOS™ FL Imaging System (Invitrogen™, Thermo Fisher Scientific). Generated forces were calculated with the following formula:


(1) $$\mathrm{Force}\;(N)=\left(\mathrm{Ew}t^{3}\right)/\left[2a^{2}(3L-a)\right]\times \delta$$

with a PDMS stiffness of 1.59 ± 0.2792 MPa, where (E) is the elastic modulus, (w) is the width of the pillar, (t) is the thickness of the pillar, (a) is the length to the attachment point of tissue with respect to the anchor point of the pillar, (L) is the length of the pillar and (δ) the displacement of the pillar.^46^

Information on RNA isolation, complementary DNA synthesis, RT-qPCR analysis, tissue sectioning and immunofluorescence staining can be found in the Supplementary material. Information on primer sets used for RT-qPCR analysis is listed in Supplementary Table 3. Information on primary and secondary antibodies used is listed in Supplementary Table 4.

### RNA sequencing and data analysis

2D myotubes and 3D-TESMs were differentiated for 4 or 14 days, respectively, after which cultures were harvested and RNA was isolated as described above.

PolyA-tailed RNA sequence libraries were generated with the NEBNext Ultra II Directional RNA Library Prep Kit for Illumina (NEB #E7760S/L) according to the manufacturer’s protocol. Samples were sequenced using a NovaSeq 6000 sequencer using paired-end 150 bp sequencing read length. Image analysis, base calling and quality checking was performed with the NextSeq 500 RTA software (v.3.4.4, Illumina)^50^ and Bcl2fastq (v.2.20, Illumina).^51^

Reads were trimmed and quality filtered by TrimGalore (v.0.4.5, cutadapt v.2.9)^52^ using default parameters and mapped to Genome Reference Consortium Human Build 38 (GRCh38, Gencode release 28)^53^ using STAR aligner (v.2.5.1b).^54^ Duplicated reads were removed from the BAM files using UMI-tools (v.1.0.1).^55^ A gene expression counts table was generated using HTSeq (v.0.11.3, genome annotation hg38).^56^ Initially, raw gene count tables were normalized using the median of ratios method internally implemented in the DESeq2 R Package (v.1.34.0).^57^ Subsequently, the DESeq2 variance stabilizing transformation was applied to normalize and log-transform gene expression values. The transformed data were used for principal component analysis.

Differential gene expression analysis was performed using DESeq2 in R for each comparison, with adjusted *P*-values reported using the Benjamini–Hochberg multiple testing correction method. Gene ontology (GO) enrichment analysis of upregulated and downregulated genes was conducted using the ClusterProfiler R package (v.4.2.2).^58^ Visualization of all RNA-sequencing data results was carried out using the ggplot2 R package (v.3.4.4).^59^

### Drug treatment of 3D tissue engineered skeletal muscles

The small molecules pamapimod (RO4402257; Bio-connect), rebastinib (MedChemExpress) and CK1 inhibitor (PF670462; Bio-connect) were dissolved in dimethyl sulphoxide (DMSO) according to the manufacturer’s instructions. Compounds were added at the concentrations indicated in the figures.

### Statistical analysis

Statistical analysis was performed using GraphPad Prism v.9.3.1 software. Statistical differences between two groups were determined by Student’s unpaired *t*-test. Statistical differences between more than two groups were determined by one-way ANOVA followed by Bonferroni multiple comparison test. All values are shown as the mean ± standard deviation (SD), with significance defined as *P* ≤ 0.05.

## Results

### Affected and non-affected myogenic progenitors show similar expansion and differentiation capacities

To generate genetically matched MPs from hiPSCs, we characterized hiPSC lines from three unrelated mosaic FSHD1 patients (Supplementary Table 1). Single colonies were picked, expanded, and screened for D4Z4 repeat array sizes using pulsed field gel electrophoresis followed by Southern blot analysis.^60^ Previously, one affected and one non-affected hiPSC clone for Patients 1 and 2 (C1.1, F1.1, C2.2 and F2.1) were generated and characterized in our laboratory.^44^ Here, we selected one additional affected and non-affected hiPSC clone for Patients 1 and 2 and added two affected and two non-affected hiPSC clones of a third mosaic FSHD1 patient for characterization (Supplementary Table 2). Pulsed field gel electrophoresis and Southern blot analysis confirmed diagnostically established FSHD1-D4Z4 repeat array sizes for all affected hiPSC lines (F1.2, F2.2, F3.1 and F3.2), when comparing with the parental mosaic fibroblasts, with sizes of three, two and three D4Z4 units, respectively (Supplementary Fig. 1). In non-affected hiPSC lines (C1.2, C2.1, C3.1 and C3.2), normal-sized D4Z4 repeat array sizes of 45, 43 and 19 units, respectively, were confirmed. Next, these eight novel hiPSC lines were tested for the presence of pluripotency markers using immunofluorescence staining. All hiPSC lines showed positive staining for Oct3/4, SSEA4 and NANOG (Supplementary Fig. 2). Furthermore, hiPSCs were differentiated spontaneously to determine the formation of cells in the three germ layers. Immunofluorescence staining showed cells expressing vimentin or NCAM (mesoderm), PAX6 (ectoderm) and FOXA2 (endoderm) for all hiPSC lines (Supplementary Fig. 3). Finally, the genomic integrity of the hiPSC lines was confirmed using Global Screening Arrays (GSA v.1, Illumina Inc.). For each single nucleotide polymorphism, the relative signal intensities were quantified, and no copy number abnormalities were detected (Supplementary Fig. 4).

Next, all hiPSC lines were differentiated into MPs using a previously published transgene-free myogenic differentiation protocol.^46,61^ For this, single hiPSCs were treated for 2 days with CHIR99021, followed by a 14-day incubation with minimal medium containing FGF and the remaining 15 days with minimal medium without FGF. Around Day 31, MPs were successfully purified by fluorescence-activated cell sorting (FACS) by selecting c-MET^+^/HNK1^−^ cells (data not shown), yielding two genetically matched pairs of non-affected and affected MPs per patient (12 lines in total; 6 affected and 6 non-affected).

**Figure 2:**
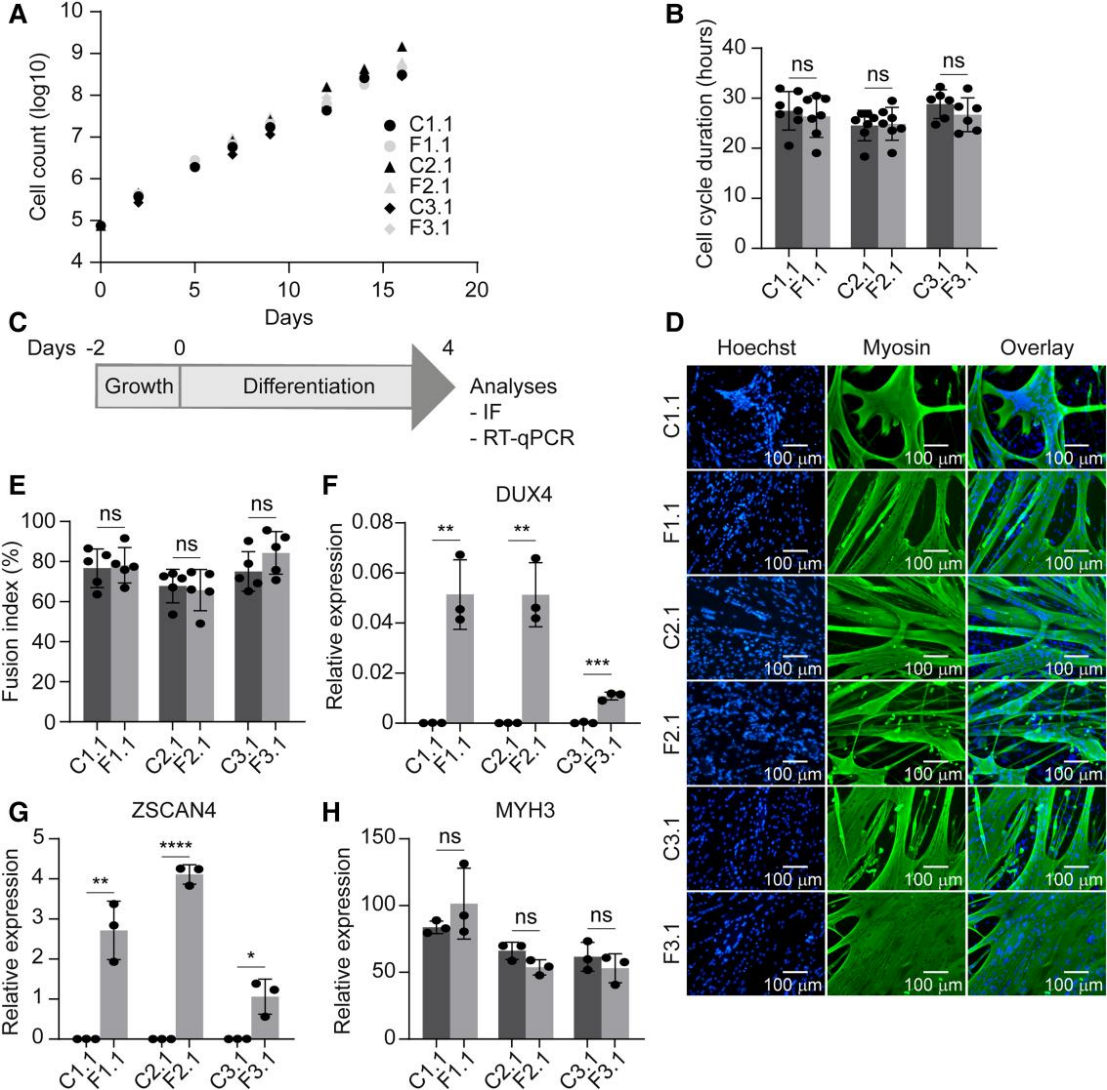
**Characterization of non-affected and affected myogenic progenitors from mosaic FSHD1 patients in 2D myotube cultures.** (**A**) Proliferation curve of genetically matched non-affected (C) and affected (F) myogenic progenitors (MPs) from Patient 1 clone 1 (C1.1 and F1.1), Patient 2 clone 1 (C2.1 and F2.1) and Patient 3 clone 1 (C3.1 and F3.1) in 2D myotube cultures. (**B**) Cell cycle duration of MPs from **A**. Each dot represents one biological replicate, and the error bars denote the standard deviation (SD). (**C**) Time line of MP differentiation in 2D myotube cultures. Cells were grown for 2 days in growth medium, after which the medium was replaced with differentiation medium containing 10 μM SB431542 (TGFβ pathway inhibitor). After 4 days of differentiation, cells were either fixed for immunofluorescence (IF) staining or harvested for RNA. (**D**) Representative immunofluorescence images of differentiated MPs. Nuclei were stained with Hoechst (blue), and myosin was stained with MF20 (green). (**E**) Quantification of fusion index [percentage fused nuclei (in myotubes) out of total amount of nuclei] after MP differentiation in 2D myotube cultures. Per cell line, five random fields were analysed. Each dot represents one random field. (**F**–**H**) Gene expression analyses of *DUX4* (**F**), *ZSCAN4* (**G**) and *MYH3* (**H**) from differentiated MPs in 2D myotube cultures using RT-qPCR. Gene expression is shown as relative expression to the housekeeping gene *GUSB*. Each dot represents one biological replicate, and the error bars denote the SD. (**B** and **E**–**H**) Statistical analysis was performed using Student’s unpaired *t*-tests. ns = not significant. *P < 0.05, **P < 0.01, ***P < 0.001, ****P < 0.0001. FSHD1 = facioscapulohumeral muscular dystrophy type 1.

Genetically matched MP pairs were next compared for their growth and differentiation kinetics in 2D culture. For this, MPs were cultured for 16 days to determine expansion capacity. Both non-affected and affected MP lines showed comparable proliferation rates (Fig. 2A and Supplementary Fig. 5A), with an average cell cycle duration of ∼28 h (Fig. 2B and Supplementary Fig. 5B). MPs were subsequently differentiated into myotubes to assess fusion indexes in 2D culture. MPs were seeded and cultured for 2 days in growth medium to reach confluency, after which MPs were kept for an additional 4 days in differentiation medium (Fig. 2C). Immunofluorescence staining for myosin confirmed differentiation of MPs into myotubes for both non-affected and affected cell lines (Fig. 2D and Supplementary Fig. 5C). Quantification of fusion indexes showed comparable fusion rates in non-affected and affected MPs (Fig. 2E and Supplementary Fig. 5D). Gene expression analysis of differentiated MPs by RT-qPCR showed *DUX4* and DUX4 target gene *ZSCAN4* expression in affected MPs only (Fig. 2F and G and Supplementary Fig. 5E and F). No significant differences in *MYH3* mRNA expression were detected between myotubes from non-affected and affected genetically matched pairs (Fig. 2H and Supplementary Fig. 5G).

Taken together, non-affected and affected MPs derived from hiPSCs from mosaic FSHD1 patients showed similar expansion and fusion characteristics in 2D culture. Furthermore, only affected MPs showed evidence for *DUX4* and DUX4 target gene expression after differentiation, with no effect on differentiation capacity.

### Non-affected and affected myogenic progenitors show comparable differentiation capacity in 3D-TESMs

Having established that 2D cultures showed similar myogenic properties between non-affected and affected MPs from mosaic FSHD1 patients, we generated a 3D-TESM model for FSHD. To this end, MPs were fused in the presence of hydrogel and cast in our previously developed 50 μl chambers fabricated by the direct peeling method.^48^ These 3D devices consist of a ‘T-bone’ made from PDMS, with two flexible pillars allowing for the attachment of myotubes and the formation of a functional skeletal muscle. This system allows contractile measurements as functional readout and culture times of ≥2 weeks.

**Figure 3:**
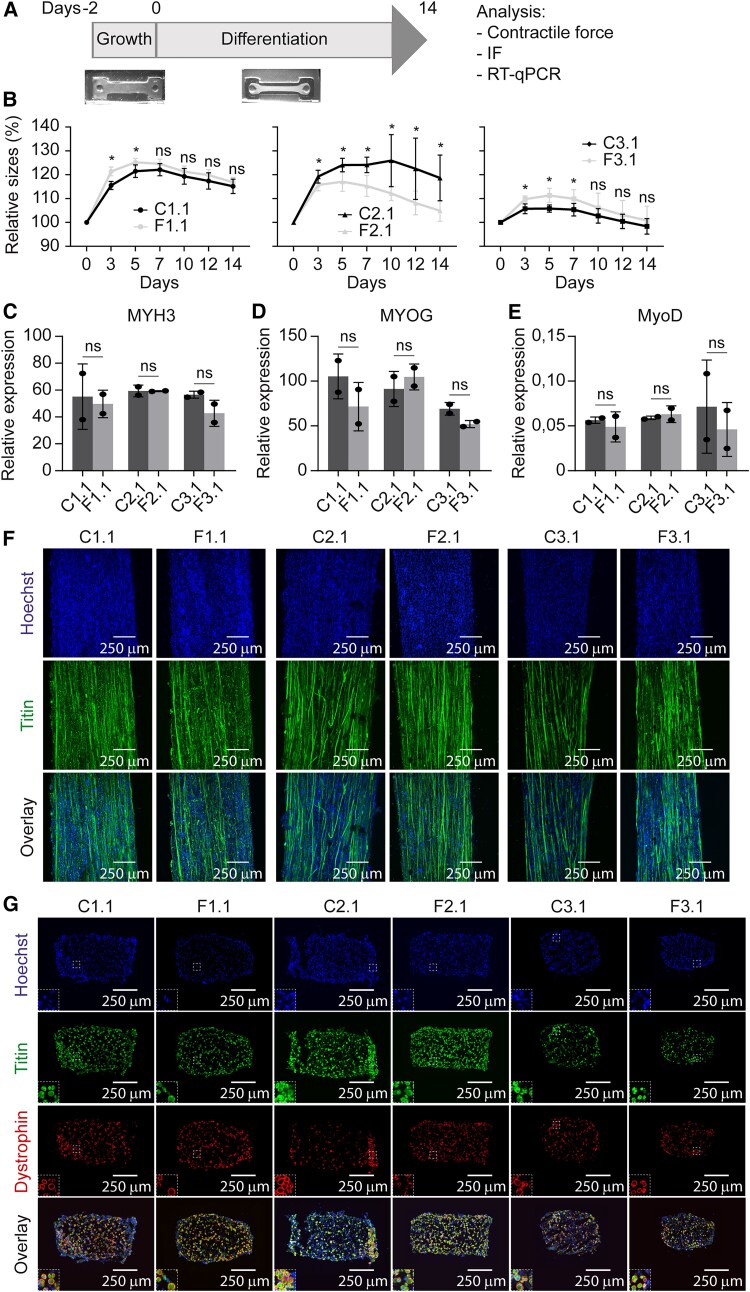
**Characterization of non-affected and affected myogenic progenitors from mosaic FSHD1 patients in 3D-TESMs differentiated for 14 days.** (**A**) Time line of myogenic progenitor (MP) differentiation into 3D tissue engineered skeletal muscles (3D-TESMs). Cells were grown for 2 days in 3D growth medium, after which the medium was changed to 3D differentiation medium supplemented with 10 μM SB431357 (TGFβ pathway inhibitor). At Day 14 of differentiation, 3D-TESMs were subjected to electrical stimulation for contractile force measurements, after which 3D-TESMs were either fixed for immunofluorescence (IF) staining or harvested for RNA. (**B**) Relative width sizes of non-affected (**C**) and affected (**F**) 3D-TESMs grown from MPs of Patient 1 clone 1 (C1.1 and F1.1), Patient 2 clone 1 (C2.1 and F2.1) and Patient 3 clone 1 (C3.1 and F3.1) over time. Statistical analysis was performed using Student’s unpaired *t*-tests. Data are shown as the average of 12 3D-TESMs per line, with error bars denoting the standard deviation (SD). Relative width sizes were normalized to Day 0 of differentiation. ns = not significant; *P < 0.05. (**C**–**E**) Gene expression analyses of *MYH3* (**C**), *MYOG* (**D**) and *MYOD* (**E**) from non-affected and affected 3D-TESMs by RT-qPCR. Gene expression is shown as relative expression to the housekeeping gene *GUSB*. Statistical analysis was performed using Student’s unpaired *t*-tests. Each dot represents one biological replicate, and the error bars denote the SD. ns = not significant. (**F**) Representative images of whole-mount immunofluorescence staining of 3D-TESMs. Nuclei were stained with Hoechst (blue), and muscle fibres were stained for titin (green). (**G**) Representative images of cross-sections from non-affected and affected 3D-TESMs. Cross-sections were stained with Hoechst (blue), anti-titin (green) and anti-dystrophin (red) antibodies. FSHD1 = facioscapulohumeral muscular dystrophy type 1.

3D-TESMs of all MP lines were generated in 12-fold and incubated for 2 days in growth medium, followed by incubation for 14 days in differentiation medium, as previously described^46^ (Fig. 3A). The formation of 3D-TESMs was monitored over 2 weeks. 3D-TESMs of all MP lines showed an increase in width from initiation of differentiation until Day 5 or 7 of differentiation, after which the width of all 3D-TESMs decreased until the end point at Day 14 of differentiation (Fig. 3B and Supplementary Fig. 6A). Gene expression analysis of the 3D-TESMs by RT-qPCR showed similar *MYH3* (embryonic form of *MYH*), *MYOG* and *MYOD* levels between non-affected and affected 3D-TESMs from each patient at Day 14 of differentiation (Fig. 3C–E and Supplementary Fig. 6B–D). We also determined the expression of *MYH8* (neonatal), *MYH7* (type 1), *MYH2* (type 2A), *MYH1* (type 2X) and *MYH4* (type 2B) in the first pairs of non-affected and affected 3D-TESMs (Supplementary Fig. 7). We found that the order of expression from high to low was *MYH8*, *MYH7*, *MYH2*, *MYH1* and *MYH4*, suggesting that the 3D-TESMs are still neonatal after 14 days of differentiation. For *MYH8* and *MYH7*, a significantly higher expression level was found in affected 3D-TESMs of Patients 2 and 1, respectively, in comparison to non-affected 3D-TESMs.

Next, to examine myofibre formation in 3D-TESMs, whole-mount immunofluorescence staining for titin was performed on tissues differentiated for 14 days. All 3D-TESMs showed multinucleated and aligned myofibres (Fig. 3F and Supplementary Fig. 6E). Cross-sectional analysis and immunostaining further revealed dystrophin and titin positivity for all 3D-TESMs (Fig. 3G and Supplementary Fig. 6F). Collectively, we found that all non-affected and affected MPs from mosaic FSHD1 patients were able to form myofibres in 3D-TESMs, with a comparable differentiation capacity.

### Reduced absolute contractile forces, thinner myofibres and DUX4 expression in affected 3D-TESMs

To assess their force-generating capacity, 3D-TESMs were stimulated electrically with a 1 Hz for 1 s (twitch) and a 20 Hz for 2 s (tetanic) pulse using a customized Arduino system. Pillar displacement, 3D-TESM height on the pillar, and PDMS stiffness were determined to calculate forces using Equation 1.

**Figure 4:**
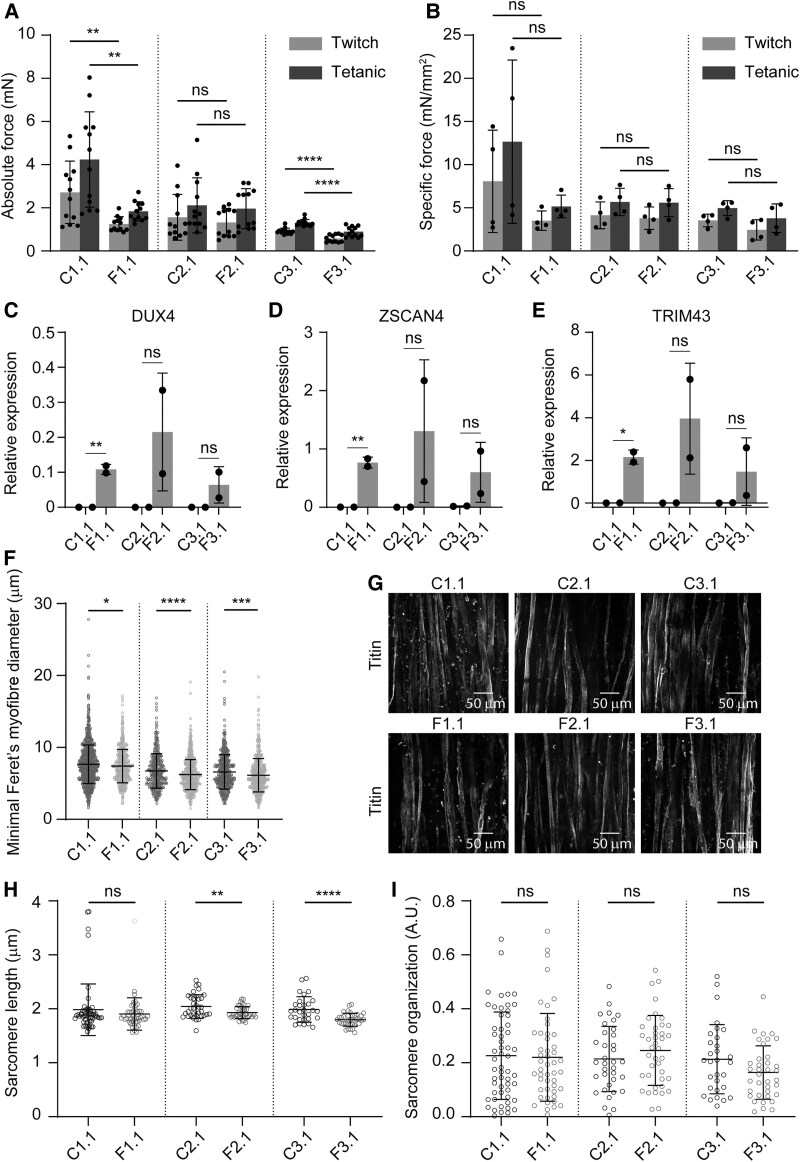
**Differences between non-affected and affected 3D-TESMs from mosaic FSHD1 patients differentiated for 14 days.** (**A**) Absolute forces of non-affected and affected 3D tissue engineered skeletal muscles (3D-TESMs) after electrical stimulation at 1 Hz (twitch; grey bars) and 20 Hz (tetanic; dark grey bars). Each dot represents one biological replicate, and the error bars denote the standard deviation (SD). (**B**) Specific forces of 3D-TESMs as in **A**, normalized for their cross-sectional area. Each dot represents one biological replicate, and the error bars denote the SD. (**C**–**E**) Gene expression analysis of *DUX4* (**C**), *ZSCAN4* (**D**) and *TRIM43* (**E**) in non-affected and affected 3D-TESMs using RT-qPCR. Gene expression is shown as relative expression to the housekeeping gene *GUSB*. Each dot represents one biological replicate, and the error bars denote the SD. (**F**) Quantification of the minimal Feret’s myofibre diameter (in μm) from myofibres stained for dystrophin in 3D-TESM cross-sections. A minimum of 450 myofibres was analysed per biological replicate (*n* ≥ 3) per cell line. Values are shown as the mean ± SD. (**G**) Representative images of whole-mount-stained 3D-TESMs at ×40 magnification. 3D-TESMs were stained for titin (white). (**H** and **I**) Quantification of sarcomere length (in micrometres) (**H**) and sarcomere organization score (in arbitrary units) (**I**) using SotaTool software. A minimum of 30 myofibres was analysed per condition from one biological replicate. (**A**–**C**, **F**, **H** and **I**) Statistical analysis was performed using Student’s unpaired *t*-tests. ns = not significant, *P < 0.05, **P < 0.01, **P < 0.01, ***P < 0.001, ****P < 0.0001. FSHD1 = facioscapulohumeral muscular dystrophy type 1.

Absolute force measurements showed significantly reduced twitch and tetanic forces in affected 3D-TESMs compared with non-affected 3D-TESMs in five of six genetically matched pairs (Fig. 4A and Supplementary Fig. 8A). Only the first genetically matched pair of Patient 2 showed no significant reduction in absolute forces. Specific forces (absolute forces normalized for the cross-sectional area) showed no significant reduction in twitch and tetanic forces in five of six genetically matched pairs (Fig. 4B and Supplementary Fig. 8B), which is caused by a reduced cross-sectional area of affected 3D-TESMs. Only the second genetically matched pair of Patient 3 showed significant differences in specific twitch and tetanic forces. In line with 2D culture, gene expression analysis of all genetically matched pairs of 3D-TESMs showed *DUX4* and DUX4 target genes *ZSCAN4* and *TRIM43* expression in affected 3D-TESMs only (Fig. 4C–E and Supplementary Fig. 8C–E).

Next, we determined the myofibre diameter (Fig. 4F and Supplementary Fig. 8F) and number of dystrophin-positive myofibres per millimetre squared (Supplementary Fig. 9) for each cell line from dystrophin-stained cross-sections. We detected significantly thinner myofibres in five of six pairs of affected 3D-TESMs in comparison to their genetically matched non-affected counterparts. In the second pair of Patient 2, no significant differences were found in myofibre sizes. There was a similar number of dystrophin-positive myofibres per millimetre squared between affected and non-affected 3D-TESMs; only the second pair of Patient 3 showed a significant difference. Finally, we used SotaTool^62^ to quantify the sarcomere organization and length in our affected and non-affected myofibres by using high-resolution whole-mount titin-staining images (Fig. 4G and Supplementary Fig. 8G). SotaTool is an image analysis software package that automatically detects the direction of the highest sarcomere organization score in an image, giving a sarcomere organization score and the sarcomere length. Quantification of titin-stained single myofibres by SotaTool revealed only for the first genetically matched pairs of Patient 2 and 3 smaller sarcomeres in affected 3D-TESMs compared with their non-affected equivalents, whereas for the second genetically matched pairs and both pairs of Patient 1 no significant differences in sarcomere length were observed (Fig. 4H and Supplementary Fig. 8H). The sarcomere organization score was significantly different between affected and non-affected single fibres only for the second genetically matched pair of Patients 1 and 3, whereas the other genetically matched pairs showed similar scores (Fig. 4I and Supplementary Fig. 8I).

Collectively, these data show reduced absolute forces in affected 3D-TESMs for five of six genetically matched pairs from three mosaic FSHD1 patients, with *DUX4* and DUX4 target gene expression in affected 3D-TESMs only. Specific forces were, however, similar for five of six genetically matched pairs, which shows that affected 3D-TESMs are smaller in size but relatively as strong as non-affected 3D-TESMs. Furthermore, thinner myofibres were observed in five of six affected 3D-TESMs, and smaller sarcomeres were found in three of six affected 3D-TESMs compared with their genetically matched controls. Affected and non-affected cell lines from the same patient thus displayed heterogeneity, as differences in contractile forces between clones were observed.

### Advanced cellular differentiation and increased DUX4 target gene expression in affected 3D-TESMs

We conducted RNA sequencing on 2D myotubes (4 days of differentiation) and 3D-TESMs (14 days of differentiation) derived from both affected and non-affected MPs of the first genetically matched pairs, aiming to obtain a more profound and comprehensive understanding of the transcriptomic changes. Principal component analysis indicated a clear segregation between 2D myotubes and 3D-TESMs along principal component 1, suggesting that the primary difference arose from distinct culture conditions and myogenic differentiation. No significant differences were observed within either the 2D myotube cultures or 3D-TESMs (Supplementary Fig. 10A).

**Figure 5:**
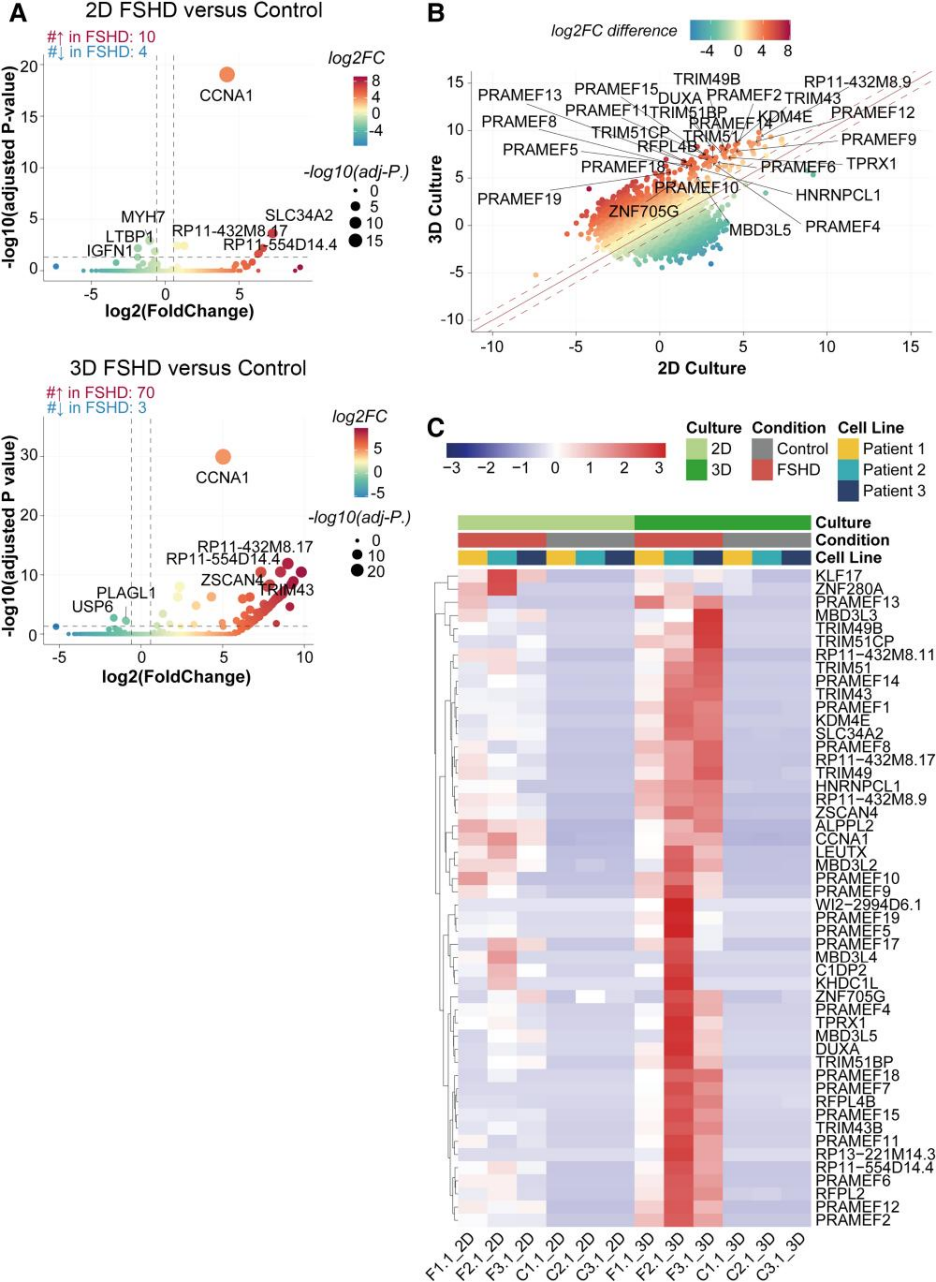
**RNA sequencing analysis from 2D and 3D cultures of non-affected and affected myogenic progenitors from mosaic FSHD1 patients.** (**A**) Volcano plots depicting the results of differential gene expression analysis by comparing affected (FSHD) and non-affected (control) samples within the 2D cultures (*top*) or 3D cultures (*bottom*). Colour scales depict the log2FoldChange (log2FC) of each gene, and the size of dots shows the value of −log10(adjusted *P*-value). Genes were classified as differentially expressed when they showed a minimum 1.5-fold expression change [|log2(FC)| > log2(1.5)] and statistical significance (adjusted *P*-value <0.05). (**B**) Scatter plot showing the log2FC difference of each gene between 2D culture and 3D culture. Colour scales depict the log2FC difference. Red represents the larger difference in log2FC in 3D culture and green represents the larger difference in log2FC in 2D culture. (**C**) Heat map showing the expression level of detected DUX4 target genes in each RNA-sequencing sample. The data were normalized in TMM using DEseq2. Colour scales depict the expression level normalized in *z*-score. -FSHD1 = facioscapulohumeral muscular dystrophy type 1.

Subsequently, we undertook differential gene expression analysis by comparing affected and non-affected samples within the 2D and 3D cultures, respectively. In the 2D myotube cultures, we identified 10 significantly upregulated genes in affected samples, including eight DUX4 target genes (Fig. 5A and Supplementary Table5).^63^ Conversely, within the 3D-TESMs, we detected more significantly upregulated genes in affected samples (*n* = 70), comprising 32 DUX4 target genes (Fig. 5A and Supplementary Table 6), aligning with previous studies linking the intensity of DUX4-associated signatures with muscle differentiation.^64^ Moreover, by calculating log2FoldChange differences between 2D MP cultures and 3D-TESMs we found higher levels of change in expression levels of the DUX4 target genes in 3D-TESMs compared with 2D myotubes (Fig. 5B). Among the 47 detectable DUX4 target genes, 43 exhibited higher fold changes in 3D-TESMs (Supplementary Fig. 10B and Supplementary Table 7). Notably, different MP lines displayed cellular heterogeneity reflected in varied expression levels and patterns of DUX4 target genes (Fig. 5C).

To evaluate directly whether FSHD-related signatures undergo more significant alterations in 3D culture, we conducted differential gene expression analysis on affected samples cultured in 2D and 3D systems. We identified 694 upregulated genes in 3D conditions, including 11 DUX4 target genes, alongside 543 significantly downregulated genes (Supplementary Fig. 10C and Supplementary Table 8). Notably, *PRAMEF2* and *TRIM43* were identified, consistently with previous studies, where both genes were considered late-stage expressed genes in a pseudo-time model constructed based on myogenesis.^65^

We next performed gene ontology (GO) enrichment analysis for biological process terms on all upregulated and downregulated genes. The upregulated genes were enriched for GO terms associated with extracellular matrix formation and myogenesis (Supplementary Fig. 10D), whereas the downregulated genes were significantly enriched in terms related to energy metabolism (Supplementary Fig. 10E and Supplementary Table 9), suggesting that the primary differences stemmed from culture conditions and differentiation rather than FSHD-related signatures. Moreover, we determined the PAX7 score, because this score is suppressed in FSHD.^66^ In 2D cultures, no significant reduction of PAX7 score was observed between affected and non-affected MPs (Supplementary Fig. 11). In 3D cultures, affected 3D-TESMs showed a reduced but non-significant PAX7 score compared with non-affected 3D-TESMs.

In summary, our RNA-sequencing data illustrated that in 3D culture conditions cellular differentiation improved, and the expression of DUX4 target genes correspondingly increased, underscoring the advantage of 3D culture over 2D culture in elucidating FSHD pathology.

### Treatment of 2D and 3D culture with small molecules

**Figure 6:**
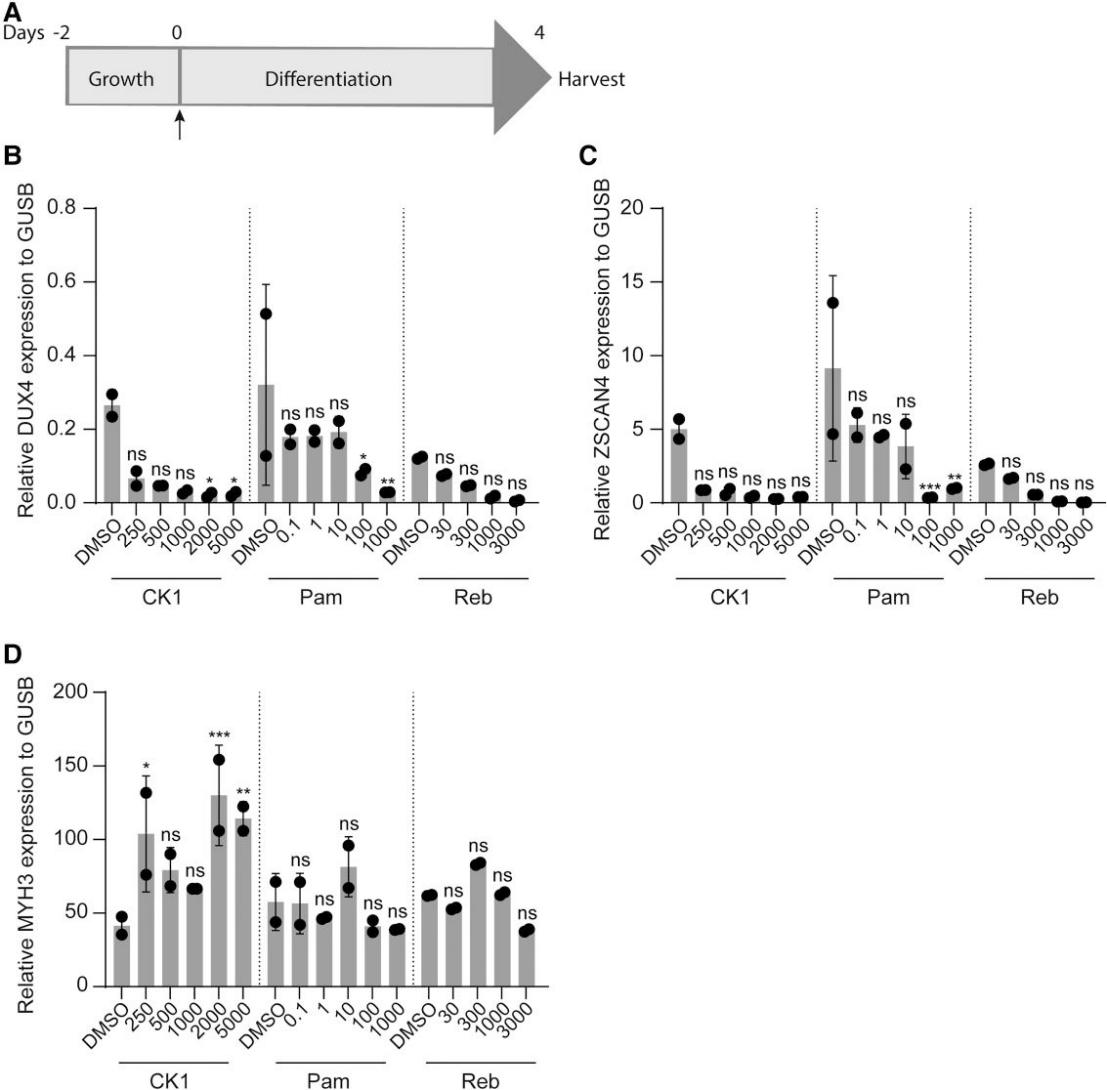
**Treatment of affected myogenic progenitors from mosaic FSHD1 patients in 2D myotube cultures with DUX4 inhibitors.** (**A**) Time line of treatment of myogenic progenitors (MPs) in 2D myotube cultures. Cells were grown for 2 days in proliferation medium, after which medium was changed to 2D differentiation medium containing DUX4 inhibitors. MPs were differentiated and treated for 4 days, after which they were harvested for RNA. (**B**–**D**) Gene expression analyses of *DUX4* (**C**), *ZSCAN4* (**D**) and *MYH3* (**E**) in differentiated MPs of Patient 2 FSHD clone 1 in 2D myotube cultures after treatment with CK1 (final concentration ranging from 250 to 5000 nM), pamapimod (Pam; final concentration ranging from 0.1 to 1000 nM) or rebastinib (Reb; final concentration ranging from 30 to 3000 nM), using RT-qPCR. Gene expression is shown as relative expression to the housekeeping gene *GUSB*. Each dot represents one biological replicate, and the error bars denote the standard deviation. Significance was determined using one-way ANOVA with Bonferroni multiple comparison correction for DMSO-treated 3D tissue engineered skeletal muscles. ns = not significant, *P < 0.05, **P < 0.01, ***P < 0.001. FSHD1 = facioscapulohumeral muscular dystrophy type 1.

Several DUX4-suppressing small molecules have been identified in 2D cell cultures, including pamapimod,^67^ an analogue of losmapimod, a small molecule that was recently tested in clinical trials as a potential treatment for FSHD,^68,69^ CK1 inhibitor (PF-670462)^70^ and rebastinib (DCC-2036).^71^ Initially, we tested these compounds in affected MPs of Patient 2 (F2.1), because this cell line had the highest DUX4 expression. For this, we treated cells with increasing concentrations of each compound in 2D myotube cultures. A single dose of each compound was given at initiation of differentiation (Fig. 6A). After myotube formation at Day 4 of differentiation, gene expression analysis was performed. Both DUX4 expression and DUX4 target gene *ZSCAN4* expression decreased in a concentration-dependent manner for each compound (Fig. 6B and C). *MYH3* mRNA levels were increased upon treatment with CK1 inhibitor, suggestive of an effect on myogenic differentiation, whereas for pamapimod and rebastinib the *MYH3* expression levels were not affected (Fig. 6D).

**Figure 7:**
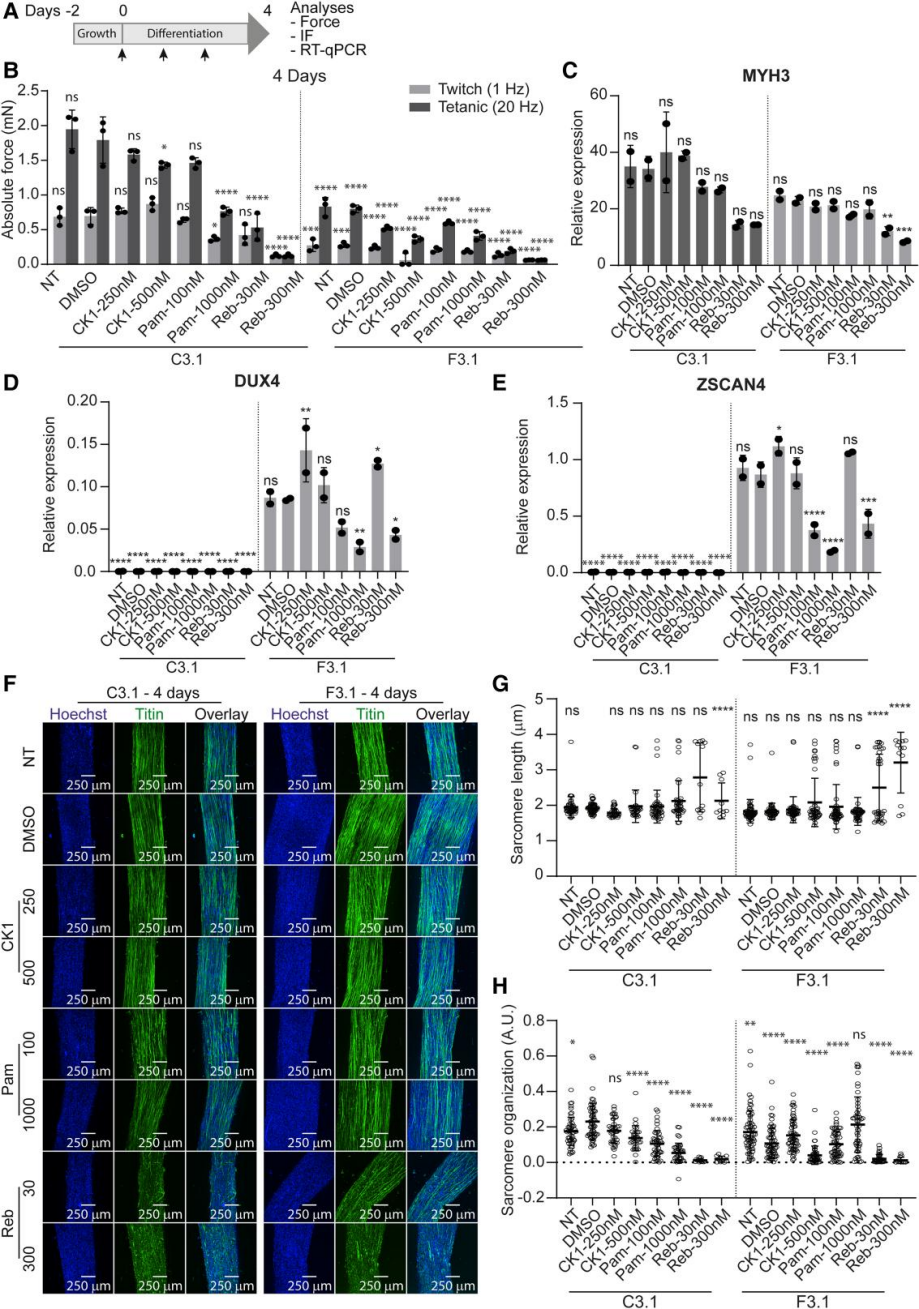
**Treatment of non-affected and affected myogenic progenitors from mosaic FSHD1 patients in 3D-TESMs with DUX4 inhibitors for 4 days.** Non-affected and affected 3D tissue engineered skeletal muscles (3D-TESMs) of mosaic FSHD1 Patient 3 (C3.1 and F3.1) were non-treated (NT) or treated daily starting at initiation of differentiation for 4 days with DMSO, CK1 inhibitor (final concentration 250 and 500 nM), pamapimod (Pam; final concentrations 100 and 1000 nM) or rebastinib (Reb; 30 and 300 nM). (**A**) Time line of treatment of 3D-TESMs. Cells were grown for 2 days in proliferation medium, after which the medium was changed to 3D differentiation medium supplemented with 10 μM SB431542 and DUX4 inhibitors. Differentiation medium containing DUX4 inhibitors was replaced every day. On Day 4 of differentiation, 3D-TESMs were subjected to electrical stimulation for contractile force measurements. Thereafter, 3D-TESMs were either fixed for immunofluorescence (IF) staining or harvested for RNA. (**B**) Absolute forces after electrical stimulation at 1 Hz (twitch; grey bars) or 20 Hz (tetanic; dark grey bars). Each dot represents one biological replicate, and the error bars denote the standard deviation (SD). (**C**–**E**) Gene expression analyses of *MYH3* (**C**), *DUX4* (**D**) and *ZSCAN4* (**E**) from treated C3.1 and F3.1 3D-TESMs using RT-qPCR. Gene expression is shown as relative expression to the housekeeping gene *GUSB*. Each dot represents one biological replicate, and the error bars denote the SD. (**F**) Representative images of whole-mount staining of treated 3D-TESMs from C3.1 and F3.1. Immunofluorescence (IF) staining was performed with Hoechst (blue) and anti-titin (green). (**G** and **H**) Quantification of sarcomere length (in μm) (**G**) and sarcomere organization score (in arbitrary units) (**H**) of single fibres from images shown in **F** using SotaTool software. For each condition, a minimum of seven fibres was analysed from one biological replicate. (**B**–**E, G** and **H**) Significance was determined using one-way ANOVA with Bonferroni multiple comparison correction for DMSO-treated non-affected (**B**, **G** and **H**) or affected (**C**–**E**) 3D-TESMs. ns = not significant. *P < 0.05, **P < 0.01, ***P < 0.001, ****P < 0.0001. FSHD1 = facioscapulohumeral muscular dystrophy type 1.

Based on the effect observed in 2D MP cultures, we next treated 3D-TESMs with two different concentrations of each compound. 3D-TESMs were treated daily from Days 0 to 4 or from Days 0 to 14 of differentiation with 250 and 500 nM of CK1 inhibitor, with 100 or 1000 nM of pamapimod, or with 30 or 300 nM of rebastinib (Fig. 7A and Supplementary Fig. 12A). On either Day 4 or Day 14 of differentiation, 3D-TESMs were stimulated electrically for absolute force measurements (Fig. 7B and Supplementary Fig. 12B), after which they were analysed with RT-qPCR for *MYH3*, *DUX4* and *ZSCAN4* expression (Fig. 7C–E and Supplementary Fig. 12C–E) and immunostained for titin to visualize the formation of myofibres (Fig. 7F and Supplementary Fig. 12F). Finally, single myofibres from titin-stained images (Supplementary Fig. 1^3^) were quantified using SotaTool for sarcomere length (Fig. 7G and Supplementary Fig. 12G) and organization (Fig. 7H and Supplementary Fig. 12H).

Table 1 summarizes all the results of the treated affected and non-affected 3D-TESMs from Fig. 7 and Supplementary Figs 1^2^ and 1,3. In short, on Day 4, affected 3D-TESMs showed no force improvement and sometimes even reduced twitch and tetanic absolute forces after treatment with tested compounds compared with DMSO-treated affected 3D-TESMs. Gene expression analysis for *DUX4* and target gene *ZSCAN4* showed a reduction only after treatment with pamapimod (1000 nM) and rebastinib (300 nM). All 3D-TESMs formed myofibres at Day 4 of differentiation, but a smaller titin-positive area was observed in rebastinib-treated affected 3D-TESMs. Sarcomere length and organization were also altered in rebastinib-treated affected 3D-TESMs. The non-affected 3D-TESMs also showed reduced contractile forces upon treatment with pamapimod (1000 nM) and rebastinib compared with DMSO-treated non-affected 3D-TESMs.

**Table 1:** Summary results of small compound treatment test

Parameter	CK1-250	CK1-500	Pam-100	Pam-1000	Reb-30	Reb-300
Non-affected 3D-TESMs, 4 days						
Twitch	0	0	0	−	−	−
Tetanic	0	0	0	−	−	−
MYH3	0	0	0	0	0	0
Titin-positive area	0	0	−	0	−	−
Sarcomere length	0	0	0	0	−	0
Sarcomere organization	0	−	−	−	−	−
Affected 3D-TESMs, 4 days						
Twitch	0	−	0	0	−	−
Tetanic	−	−	−	−	−	−
DUX4	−	0	0	+	−	+
ZSCAN4	−	0	+	+	0	+
MYH3	0	0	0	0	−	−
Titin-positive area	0	+	0	−	−	−
Sarcomere length	0	0	0	0	−	−
Sarcomere organization	0	−	0	−	−	−
Non-affected 3D-TESMs, 14 days						
Twitch	0	−	0	−	−	−
Tetanic	0	−	0	−	−	−
MYH3	0	0	0	0	0	0
Titin-positive area	−	−	−	−	−	−
Sarcomere length	0	0	0	0	0	−
Sarcomere organization	0	0	0	0	0	0
Affected 3D-TESMs, 14 days						
Twitch	0	−	−	−	−	−
Tetanic	−	−	−	−	−	−
DUX4	0	0	0	0	−	−
ZSCAN4	0	0	0	0	−	−
Titin-positive area	0	−	−	−	−	−
Sarcomere length	0	0	0	0	0	−
Sarcomere organization	0	0	0	0	−	−

Compound-treated [CK1 inhibitor (CK1), pamapimod (Pam) and rebastinib (Reb)] 3D tissue engineered skeletal muscles (3D-TESMs) were compared with DMSO-treated 3D-TESMs. ^+^improvement; ^−^decline; ^0^no change. Pam = pamapimod; Reb = rebastinib.

On Day 14 of differentiation, twitch and tetanic absolute forces in treated affected 3D-TESMs also showed no improvement or a decline compared with DMSO-treated affected 3D-TESMs. In addition, treatment of affected 3D-TESMs with each of the small molecules did not significantly reduce *DUX4* and *ZSCAN4* expression. A smaller titin-positive area was observed in all treated affected 3D-TESMs compared with DMSO-treated affected 3D-TESMs. Finally, sarcomere length and organization were altered in rebastinib-treated affected 3D-TESMs. Like affected 3D-TESMs, the non-affected 3D-TESMs also showed reduced contractile forces upon treatment with CK1 inhibitor, pamapimod and rebastinib in comparison to DMSO-treated non-affected 3D-TESMs.

Overall, the small molecules CK1 inhibitor, pamapimod and rebastinib showed significant reduction of *DUX4* expression levels in 2D myotube cultures. In 3D-TESMs, however, these small molecules did not improve absolute contractile forces in both non-affected and affected 3D-TESMs and showed minimal to no reduction in DUX4 expression upon daily treatment. Thus, the small molecules tested here showed a general negative effect on muscle functionality when used daily for 4 or 14 days, in both non-affected and affected 3D-TESMs.

## Discussion

In this study, we describe a 3D-TESM model for FSHD using human iPSC-derived MPs from FSHD1 patients who are somatic mosaics for the D4Z4 repeat array contraction. 3D-TESMs allowed increased culture times, myofibre alignment, modest maturation and contractile force measurements. We identified absolute force differences between non-affected and affected 3D-TESMs in five of six genetically matched pairs from three different mosaic FSHD1 patients. Furthermore, RNA sequencing revealed a stronger DUX4 transcriptome signature in affected 3D-TESMs compared with 2D myotube cultures. Although we confirmed that treatment with previously identified DUX4 inhibitors (CK1 inhibitor, pamapimod and rebastinib) reduced *DUX4* expression in 2D myotube cultures, we observed only modest effects on *DUX4* expression in 3D-TESMs and no improvement in contractile forces that were often combined with worsening of 3D-TESM functionality. Together, these data show the utility of 3D myogenic cultures in better understanding of FSHD pathophysiology by linking molecular features of the disease with functional outcomes. Its potential use in preclinical testing in drug development for FSHD, however, needs further investigation.

Although studies of human cells and biopsies have contributed to our understanding of diseases, biological variables, such as genetic and tissue heterogeneity of individuals, can strongly impact the outcome of these studies. In FSHD, family origin is an important contributor to gene expression patterns and stress responses in myogenic cell cultures.^25^ In this study, we generated human iPSC-derived MPs from mosaic FSHD1 patients to overcome some of these limitations. These MPs are genetically matched, except for the D4Z4 repeat array, which is normal in sized in control (non-affected) cell lines and contracted in FSHD (affected) cell lines. Hence, this allowed us to investigate the direct effect of DUX4 expression on muscle cells without genomic variability other than the D4Z4 repeat size.

Characterization of non-affected and affected genetically matched MPs in monolayer cultures revealed comparable biological characteristics between genetically matched pairs. Differentiation of MPs into myotubes revealed similar fusion indexes and *MYH3* levels between genetically matched affected and non-affected MPs. Fusion indexes and myogenic expression levels at Day 5 of differentiation were also similar in genetically matched immortal muscle cell clones from a mosaic FSHD1 patient^72^ and FSHD and control human myoblasts.^73^ We detected *DUX4* and *ZSCAN4* expression in affected myotubes only. This shows that after reprogramming of hiPSCs these cells retain the FSHD-specific characteristics, including derepression of *DUX4*. Overall, non-affected and affected MPs showed similar behaviour in 2D cultures, with *DUX4* expression signatures in affected cell lines only.

Symptom onset in most individuals with FSHD is around the age of 20 years,^74,75^ with ∼5%–10% of cases having an infantile onset.^4,76^ This means that skeletal muscle fibres have completely formed and matured when the first symptoms arise. In agreement, our 3D-TESMs also displayed full myofibre formation in affected cell lines after 14 days of differentiation. With RT-qPCR analysis, we detected similar gene expression levels of *MYH3*, *MYOG* and *MYOD* between genetically matched non-affected and affected 3D-TESMs. Moreover, RT-qPCR analysis of the other *MYH* revealed that the order from highest to lowest expression levels was *MYH8*, *MYH7*, *MYH2*, *MYH1* and *MYH4* for both non-affected and affected 3D-TESMs. This suggests that the 3D-TESMs still have a neonatal signature. As in 2D myotube cultures, affected MPs were able to differentiate into myofibres indifferently from non-affected MPs.

In FSHD1, contraction of the D4Z4 macrosatellite repeat on chromosome 4q leads to sporadic expression of *DUX4*, resulting in skeletal muscle weakening and cell death.^4,77^ In our system, affected 3D-TESMs displayed reduced absolute contractile forces after 14 days of differentiation in both genetically matched pairs from Patients 1 and 3, and in the second pair from Patient 2. Like 2D myotube cultures, *DUX4* was expressed only in affected 3D-TESMs. This suggests that *DUX4* expression is associated with the reduced absolute forces found in affected 3D-TESMs. We do, however, observe differences in absolute contractile forces between independent clones from the same patient (interclonal variation). We used a transgene-free protocol to differentiate hiPSCs into MPs.^45^ During differentiation, variability in epigenetic remodelling might cause biological differences between MP cultures derived from independent hiPSCs. To reduce variation in outcome, a non-affected and affected hiPSC line from the same mosaic patient were differentiated simultaneously, FACS sorted for MPs, expanded, and used for generating 3D-TESMs.

DUX4 is a transcription factor activating early developmental genes.^16,63^ To determine the transcriptomic changes in our myogenic progenitors, RNA sequencing was performed on affected and non-affected 2D myotube cultures and 3D-TESMs of the first genetically matched pairs. We detected a more extensive DUX4 signature defined by the expression of more DUX4 target genes and higher expression levels of these in affected 3D-TESMs compared with 2D myotube cultures.^63^ Using differential gene expression analysis and GO biological process term enrichment analysis, 3D cultures showed late-stage expressed genes in an earlier developed pseudo-time model constructed based on myogenesis,^65^ combined with upregulated extracellular matrix formation and myogenesis. These results show us that 3D-TESMs have increased DUX4 target gene expression, owing to improved cellular differentiation, and might have an advantage over 2D cultures when studying FSHD pathology.

Muscle pathology in FSHD biopsies consists of atrophic and regenerating muscle fibres that vary in size.^4,78,79^ Next to reduced absolute forces, thinner myofibres were observed in affected 3D-TESMs compared with non-affected 3D-TESMs in all genetically matched pairs, except in pair 2 of Patient 2. This could indicate that the myofibres in affected 3D-TESMs were atrophic, because DUX4 expression has been reported to cause atrophic myotubes in immortalized myoblasts.^80^ Alternatively, this could indicate that myofibres in affected 3D-TESMs were regenerating, which has been shown in FSHD muscle biopsies.^81^ In rats, regenerating skeletal muscle fibres produce less maximum force (∼10%) in comparison to non-regenerating muscle fibres,^82^ which might explain the lower absolute forces seen in affected 3D-TESMs compared with non-affected 3D-TESMs. When absolute forces were normalized for cross-sectional area, however, similar specific forces for affected and non-affected 3D-TESMs were found in five of six genetically matched pairs. Specific forces of single myofibres isolated from human biopsies were previously found to be preserved in FSHD,^83^ and this thus also seems to be the case in the affected 3D-TESM model used here. This suggests that there is no disease-specific contractile issue in FSHD. However, measurements of voluntary maximum force generation in patients with FSHD showed that quadriceps specific force is reduced in patients independent of disease severity or fatty infiltration.^84^ In addition, we cannot rule out that specific forces might change with increased culture times (>2 weeks). In conclusion, further investigation is required to establish whether a force defect exists in FSHD.

Currently, there is no treatment for FSHD. Small molecules that inhibit *DUX4* expression have been developed, including pamapimod, CK1 inhibitor and rebastinib that were tested in the present study. These small molecules have been shown to reduce *DUX4* expression in 2D myotube cultures, which we confirmed in the present study. p38 mitogen-activated protein kinase inhibitors, such as pamapimod, and CK1 inhibitor have also been shown to decrease *DUX4* expression levels in xenograft mice.^67,85^ Finally, an analogue of pamapimod, losmapimod, was recently tested in a phase 3 clinical trial for treating patients with FSHD1.^86^ During the phase 1 and 2 clinical trials, losmapimod was administered once or twice daily (15 mg for 14 days, phase 1 trial; or 15 mg for 48 weeks, phase 2 trial).^68,69^ Within the muscles, 63.6 ± 34 ng of losmapimod per gram of muscle was detected, which is ∼270.5 nmol losmapimod per litre of muscle (assuming that the muscle density is ∼1040 g/l). In the present study, we used pamapimod concentrations of 100 or 1000 nM for 3D-TESM treatment. We used pamapimod instead of losmapimod for treatment of 3D-TESMs, because we had previously observed that losmapimod significantly delayed differentiation of myoblasts into myotubes in 2D cultures, whereas pamapimod did not seem to have a major effect on differentiation (data not shown). We decided to administer small molecules daily from Day 0 to Day 4 or from Day 0 to Day  14 of differentiation. However, at both Day  4 and Day 14 of differentiation, none of the treated affected 3D-TESMs showed improved absolute contractile forces, myofibre density or sarcomere organization and length, in comparison to vehicle-treated affected 3D-TESMs. Moreover, using this dosing regimen small molecules seemed to have a negative effect on muscle functionality in both non-affected and affected 3D-TESMs, readouts which we were not able to observe in 2D cultures, showing the potential benefit of a 3D model over 2D cultures. Only at the highest concentrations, pamapimod and CK1 inhibitor were able to reduce *DUX4* and *ZSCAN4* levels in affected 3D-TESMs after 4 days of treatment. Thus, in our system, none of the small molecules tested showed an improvement of affected 3D-TESMs.

We could test even higher concentrations of small molecules to ensure significant *DUX4* downregulation, but these concentrations would probably not be relevant for humans. A better alternative would be to test small molecules at a later time point to ensure complete development of the 3D-TESM before exposure to compounds that potentially affect muscle formation. In addition, our 3D-TESMs have a developmental and neonatal myosin signature, and we do not know the effect of the small molecules on this stage of development. Given that none of the small molecules tested here is already approved for FSHD treatment, it is difficult to draw conclusions about the suitability of the 3D-TESM model for preclinical drug testing. Fulcrum Therapeutics recently announced that its phase 3 REACH trial for losmapimod did not achieve its primary and secondary end points and suspended further development.^87^ Given that we observed that its analogue, pamapimod, affected muscle formation, our 3D-TESM model might thus add rigour to preclinical drug development programmes.

A limitation of the present 3D-TESM model is the lack of other cell types, such as immune cells, endothelial cells and fibroadipogenic progenitors, which are normally present in skeletal muscle. These cell types might contribute to the uptake of small molecules by the 3D-TESMs, especially to the core of the 3D-TESMs. Thus, the uptake of small molecules from the culture media might have been limited in our studies, which might explain the low reduction of *DUX4* expression observed after treatment and the differences compared with results from 2D cultures and xenograft models.^67^ Of note, in the phase 2 clinical trial, losmapimod showed no changes in DUX4-driven gene expression after 48 weeks of treatment.^68^

## Conclusion

In summary, we have developed a 3D tissue engineered skeletal muscle model for FSHD1. In comparison to 2D cultures, this system shows muscle pathology that more closely resembles the pathology seen in patients with FSHD, including muscle weakness, *DUX4* expression and downstream events of DUX4. Furthermore, our data show the value of using genetically matched pairs from mosaic FSHD1 patients with a similar genetic background. We believe that the 3D-TESM model might help preclinical testing for functional studies in FSHD and in the future for development of FSHD therapy.

## Supplementary Material

awae379_Supplementary_Data

## Data Availability

Data have been uploaded to the EGA database (EGA study: EGAS50000000502).
