# Supplementary material for: Three-dimensional tissue engineered skeletal muscle modelling facioscapulohumeral muscular dystrophy
Source: Brain. 2024 Nov 18;148(5):1723–39. doi: 10.1093/brain/awae379 (PMC12074006; doi:10.1093/brain/awae379)
Supplement: awae379_Supplementary_Data [file awae379_supplementary_data.zip › brain-2024-01708-File010.pdf]

Supplementary Materials and methods

Generation of human induced pluripotent stem cells

Generation of human induced pluripotent stem cell (hiPSC) lines from mosaic fibroblasts by the Leiden hiPSC Centre was performed following a protocol previously described[1]. Briefly, fibroblasts were reprogrammed using the ReproRNA™-OKSGM kit (STEMCELL Technologies, Cologne, Germany) according to the manufacturer's instructions. Single hiPSC colonies were picked and expanded in mTESR1 medium (STEMCELL Technologies, Cologne, Germany). Confluent cultures were passaged using ReLeSR™ (STEMCELL Technologies, Cologne, Germany) onto Matrigel-coated plates (Corning, VWR, Amsterdam, the Netherlands). Each hiPSC line was checked for D4Z4 repeat array sizes using pulsed field gel electrophoresis followed by Southern blot analysis as previously described.[2] Information of clone numbers and iPSC official names are listed in **Supplementary Table 2**. Chromosomal abnormalities in hiPSCs were checked using Global Screening Array (GSA v1 Illumina Inc.) according to standard protocols, followed by analysis in GenomeStudio Software (Illumina). For spontaneous differentiation, hiPSC lines were cultured on Matrigel-coated glass coverslips (Corning, VWR, Amsterdam, the Netherlands) using the STEMdiff™ Trilineage Differentiation Kit (STEMCELL Technologies, Cologne, Germany). Cells were either fixed after 5 days (endoderm and mesoderm) or 7 days (ectoderm) with 2% paraformaldehyde (PFA; Sigma-Aldrich, Amsterdam, the Netherlands) for 30 minutes at room temperature and subsequently used for immunofluorescence staining.

Differentiation of hiPSCs to myogenic progenitors

hiPSC-derived myogenic progenitors (MPs) were generated using a previously published protocol.[3] Briefly, after expansion, hiPSCs were subjected to a 31-day myogenic differentiation protocol by first switching to myogenic differentiation medium (DMEM/F12, 1% Insulin-Transferring-Selenium-Ethanolamine (ITS-X), 1% penicillin/streptomycin/L-glutamine (P/S/G), (all Gibco, Waltham, MA, United States), supplemented with 8 µM CHIR99021 (Axon Medchem, Groningen, the Netherlands) for 2 days. For the next 14 days, cells were kept in myogenic differentiation medium supplemented with 20 ng/ml FGF2 (Prepotech, Cranbury,

NJ, United States), after which the cells were cultured without FGF2 for another 16 days. At day 31, cells were stained with anti-C-MET-APC (1:50; R&D systems, Minneapolis MN, United States), anti-HNK-1-FITC (1:100; Arviv Systems Biology, San Diego, CA, United States) antibodies, Hoechst (33258; Life Technolgies, Carlsbad, CA, United States) or Zombie Green™ Fixable Viability kit (Biolegend, San Diego, CA, United States). Subsequently, the c-MET^{+}/Hoechst^{-}/Hnk-1^{-} fraction was FACS-sorted into MP proliferation medium (DMEM high glucose (Gibco, Waltham, MA, United States) supplemented with 10% fetal bovine serum (Hyclone, Thermo Scientific, Waltham, MA, United States), 1% penicillin/streptomycin (P/S) (Gibco, Waltham, MA, United States) and 100 ng/ml FGF-2 (Prepotech, Cranbury, NJ, United States)) supplemented with 1x Revitacell supplement (Gibco, Waltham, MA, United States). After sorting, cells were seeded on ECM (E6909; Sigma-Aldrich, Amsterdam, the Netherlands)-coated dishes.

Myogenic progenitors culture

MPs were cultured on ECM-coated (1:200; E6909; Sigma-Aldrich, Amsterdam, the Netherlands) 100 mm dishes (CELLSTAR; Greiner Bio-One, Alphen aan den Rijn, the Netherlands) in MP proliferation medium. Cells were passaged using TrypLE reagent (Gibco, Waltham, MA, United States) diluted 1:1 with PBS (Gibco, Waltham, MA, United States) and kept in a humidified environment of 37 °C and 5% CO_2.

Differentiation of myogenic progenitors into myotubes

MPs were seeded on ECM-coated plates and cultured in MP proliferation medium for 2 days. Upon reaching 90% confluency, medium was switched to 2D differentiation medium (DMEM HG, 1% Penicillin-G (Sigma-Aldrich, Amsterdam, the Netherlands), 1% ITS-X and 1% knockout serum replacement (KOSR; Gibco, Waltham, MA, United States) supplemented with 10 µM SB431542 (S1067; Selleckchem, Cologne, Germany). After 4 days of differentiation, cells were fixed for immunofluorescence staining or harvested for RNA isolation.

RNA isolation, cDNA synthesis and RT-qPCR

After contractile experiments, 3D-TESMs were washed once in PBS, snap-frozen in liquid nitrogen, and stored at -80 °C. Subsequently, RNA was extracted using the miRNeasy mini kit including a DNase step according to the manufacturer's instructions (Qiagen, Venlo, the Netherlands). cDNA synthesis of equal amounts of RNA was performed using the RevertAid^{TM} H minus First Strand cDNA Synthesis Kit (Thermo Fisher Scientific, Landsmeer, the Netherlands). For RT-qPCR analysis, cDNA was diluted 10x in RNase free water, mixed with 7.5 µl SybrGreen (Bio-Rad, Veenendaal, the Netherlands) and 10 pmol of both forward and reverse primers, and analyzed on the CFX 96 or CFX 384 machine (Bio-Rad, Veenendaal, the Netherlands). Primers used are listed in **Supplementary Table 3**.

Immunofluorescence staining

Cells or 3D-TESMs were fixed in 2% or 4% paraformaldehyde (PFA; Sigma-Aldrich, Amsterdam, the Netherlands), respectively, for 1 hour at room temperature and washed with PBS for three times. For 2D staining, fixed cells were permeabilized in 0.1% Triton-X in PBS for 10 minutes, washed once with PBS, and incubated in 3% BSA and 0.1% Tween-20 in PBS for 30 minutes. Cells were than washed with PBS and incubated with primary antibodies in 0.1% BSA and 0.1% Tween-20 in PBS for 1 hour at room temperature. After incubation, cells were washed once with 0.1% Tween-20 in PBS and once in PBS for 2 minutes each. Secondary antibodies and Hoechst were diluted in 0.1% BSA and 0.1% Tween-20 in PBS and were then added to the cells and incubated for 30 minutes at room temperature in the dark. Cells were next washed with 0.1% Tween-20 in PBS and once in PBS for 2 minutes each and stored in PBS at 4 °C before imaging.

For whole mount immunostaining, fixed 3D-TESMs were permeabilized in 0.3% Triton-X, 3% BSA, and 0.1% Tween-20, all in PBS, on agitation for 1 hour at room temperature. Next, cells were washed in PBS and incubated with primary antibodies in 0.1% Triton-X, 0.1% BSA, and 0.1% Tween-20 in PBS for 1 hour at room temperature. Subsequently, 3D-TESMs were washed in 0.1% Tween-20 in PBS for 2 minutes followed by 2 minutes in PBS. Secondary antibodies and Hoechst nuclear staining were then added to the 3D-TESMs and incubated for 30 minutes at room temperature in the dark. Finally, 3D-TESMs were washed with PBS-0.1% Tween and stored in PBS at 4 °C before imaging. Cells and 3D-TESMs were imaged using the Andor Spinning disc confocal microscope Dragonfly 200 (Oxford Instruments, Oxford, United

Kingdom). Primary and secondary antibodies used in this study are listed in **Supplementary Table 4**.

Tissue sectioning of 3D tissue engineered skeletal muscles

Fixed 3D-TESMs were cut in half, removed from the chamber, and incubated for 24 hours in 30% sucrose solution (Sigma-Aldrich, Amsterdam, the Netherlands) in PBS at 4 °C. Subsequently, 3D-TESMs were embedded in O.C.T.-filled (Avantor, VWR, Amsterdam, the Netherlands) plastic cryomolds (both Tissue-Tek, Sakura, CA, United States) and snap frozen in a bath of isopentane (Sigma-Aldrich, Amsterdam, the Netherlands) chilled in liquid nitrogen, and stored at -80 °C until sectioning. 3D-TESMs were cut in 20 μm thick sections using the cryostat Leica 3050S (Leica Biosystems, Deer Park, IL, United States) chilled at -20 °C. Glass slides with cryosection slices were stored at -20 °C prior to staining.

For immunostaining, glass slides were incubated for 24 hours at room temperature after removal from -20 °C and rehydrated two times for 30 minutes in PBS at room temperature. Antigen-retrieval was performed by boiling glass slides in Tris-EDTA (10 mM Tris, 1.25 mM EDTA; pH9) buffer for 15 minutes. Slides were then washed once with PBS for 5 minutes at room temperature and then blocked in blocking solution consisting of 5% milk powder (Sigma-Aldrich, Amsterdam, the Netherlands) in 0.05% Tween20 in PBS for 10 minutes at room temperature. Next, sections were incubated with primary antibodies against Mouse IgM-anti-titin (1:50; 9D-10S, DSHB, University of Iowa, IA, United States) and dystrophin (1:100; ab15277; Abcam, Cambridge, United Kingdom) diluted in blocking solution overnight at 4 °C. Glass slides were washed three times in 0.05% Tween20 in PBS for 10 minutes, after which the slides were incubated with secondary antibodies Goat-anti-Mouse IgM Alexa fluor 488 and Donkey-anti-Rabbit Alexa fluor 594 (both 1:500; Thermo Fisher Scientific, Landsmeer, the Netherlands) and Hoechst (1:10,000; H3569; Thermo Fisher Scientific, Landsmeer, the Netherlands) for 1 hour in the dark at room temperature. Finally, tissues were washed once with PBS, dried, and a cover slide was mounted with Prolong antifade mountant (Thermo Fisher Scientific, Landsmeer, the Netherlands). Slides were stored at 4 °C before being imaged using the Andor Spinning disc confocal microscope Dragonfly 200 (Oxford Instruments, Oxford, United Kingdom).

Supplementary Figures

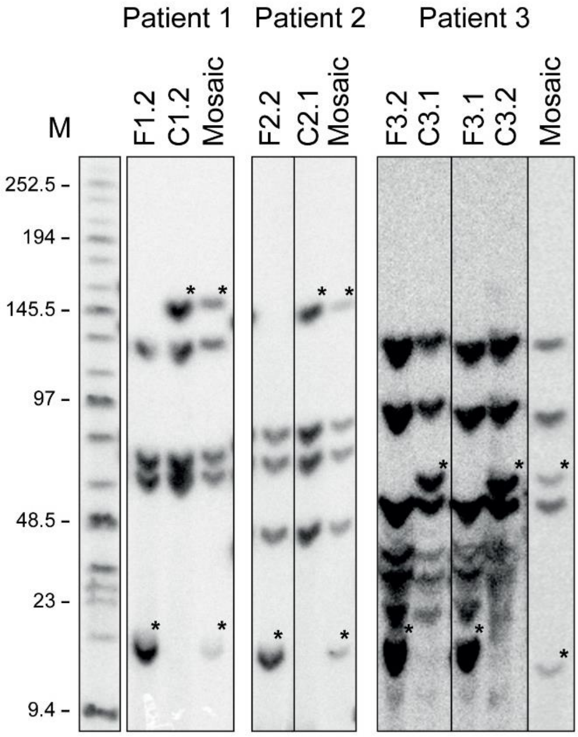

Supplementary Figure 1 Analysis of the D4Z4 repeat array in non-affected and affected human induced pluripotent stem cells (hiPSCs) and parental fibroblasts from three mosaic FSHD1 patients, using pulsed-field electrophoresis and Southern blotting. The D4Z4 repeat arrays on chromosomes 4 and 10 from affected (F), non-affected (C) hiPSCs and mosaic fibroblasts (Mosaic) from patients 1, 2, and 3 were visualized with the p13E11 probe after double digestion of genomic DNA with *Eco*RI and *Hind*III. Representative Southern blot images are shown, with the mosaic chromosome 4 bands in the mosaic fibroblasts and the different chromosome 4 bands in the affected and non-affected hiPSCs indicated by an asterisk. The molecular weight marker (M) is shown on the left side of the blots, and fragment sizes are indicated in kilobases.

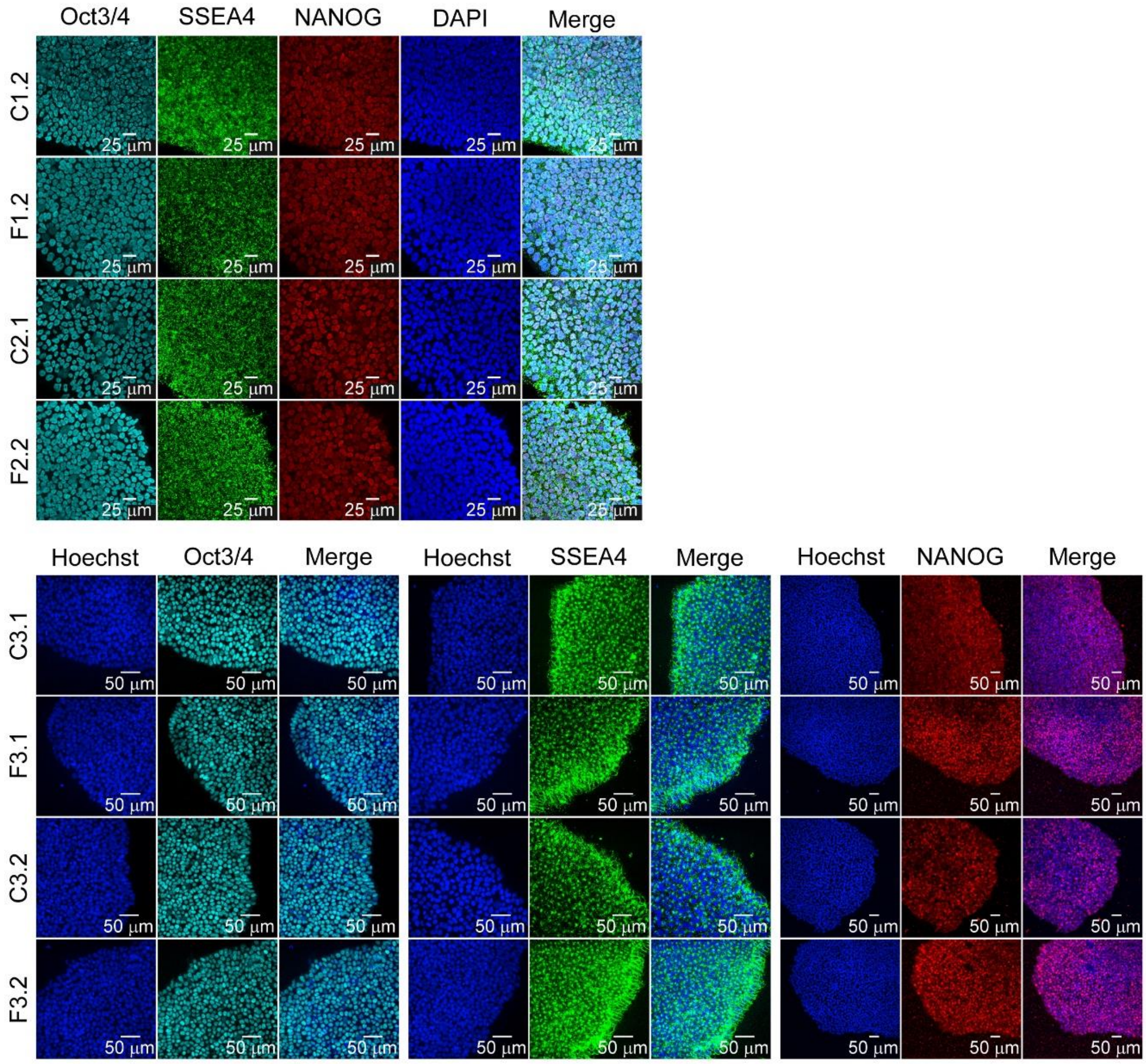

Supplementary Figure 2 Staining for pluripotency markers in non-affected and affected human induced pluripotent stem cells from mosaic FSHD1 patients. Non-affected (C) and affected (F) hiPSCs from patient 1 clone 2 (C1.2 and F1.2), patient 2 clone 1 (C2.1 and F2.2), patient 3 clone 1 (C3.1 and F3.1), and patient 3 clone 2 (C3.2 and F3.2) were stained for Oct3/4 (cyan), SSEA4 (green), and NANOG (red); nuclei were stained with either DAPI or Hoechst (blue).

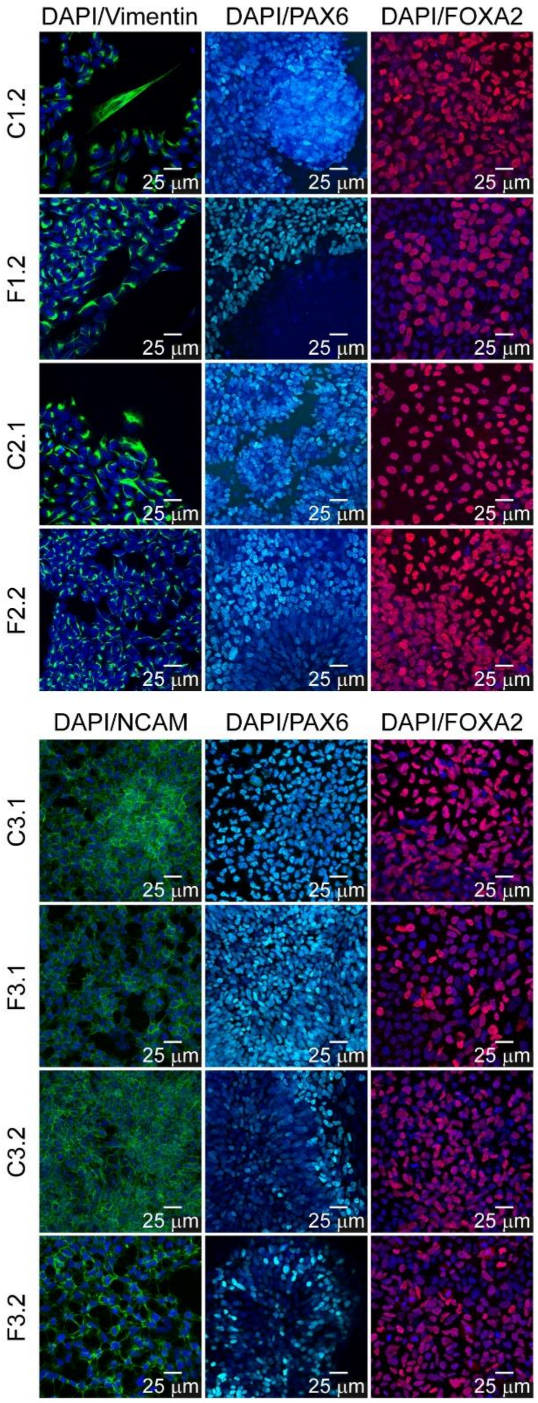

Supplementary Figure 3 Staining for differentiation markers in non-affected and affected human induced pluripotent stem cells from mosaic FSHD1 patients. Non-affected (C) and affected (F) hiPSCs from patient 1 clone 2 (C1.2 and F1.2), patient 2 clone 1 (C2.1 and F2.2), patient 3 clone 1 (C3.1 and F3.1), and patient 3 clone 2 (C3.2 and F3.2) were stained for Vimentin or NCAM (green), PAX6 (cyan), and FOXA2 (red); nuclei were stained with either DAPI or Hoechst (blue).

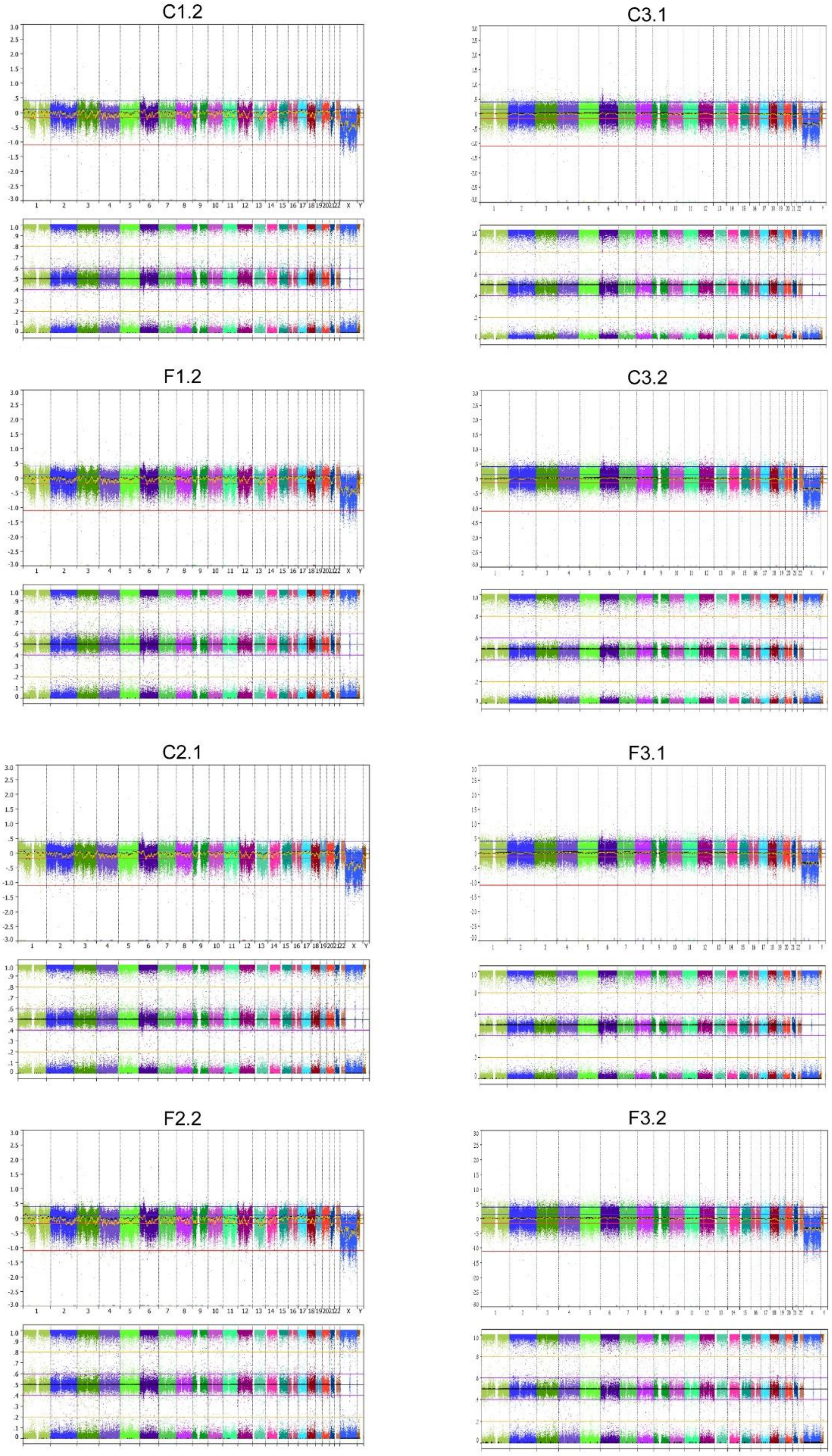

Supplementary Figure 4 SNP array analysis of non-affected and affected human induced pluripotent stem cells from mosaic FSHD1 patients. Genome-wide copy number analysis

of 700k single nucleotide polymorphisms of non-affected (C) and affected (F) hiPSCs from patient 1 clone 2 (C1.2 and F1.2), patient 2 clone 1 (C2.1 and F2.2), patient 3 clone 1 (C3.1 and F3.1), and patient 3 clone 2 (C3.2 and F3.2) using the Global Screening Array.

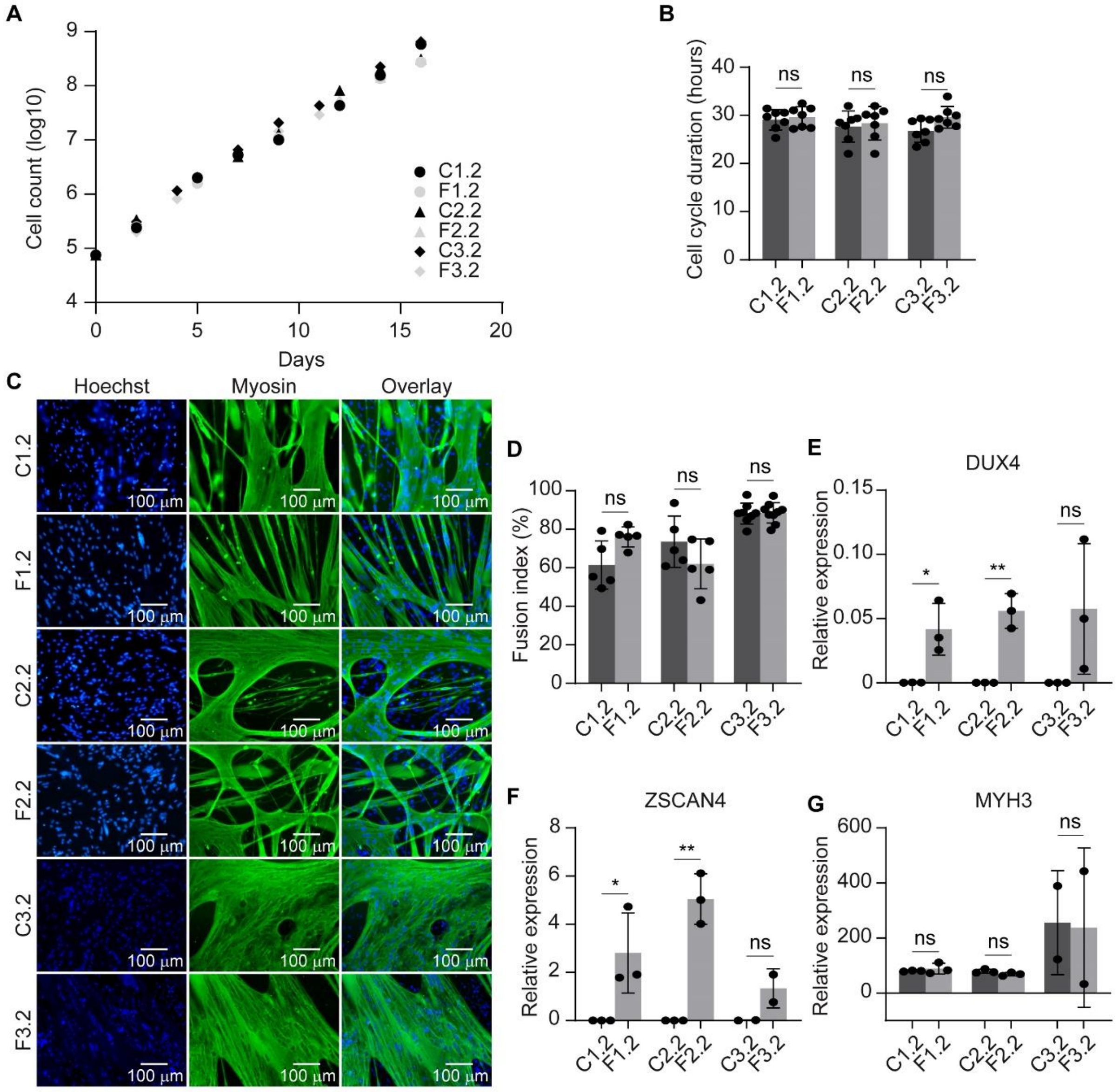

Supplementary Figure 5 Characterization of the second pair of non-affected and affected myogenic progenitors from mosaic FSHD1 patients in 2D myotube cultures.

(A) Proliferation curve of non-affected (C) and affected (F) MPs from patient 1 clone 2 (C1.2 and F1.2), patient 2 clone 2 (C2.2 and F2.2), and patient 3 clone 2 (C3.2 and F3.2). **(B)** Cell cycle duration of MPs from A. Each dot represents one biological replicate and the error bars denote the standard deviation (SD). **(C)** Representative immunofluorescence images of differentiated MPs into myotubes. Nuclei were stained with Hoechst (blue) and myosin was stained with MF20 (green). **(D)** Quantification of fusion index (percentage fused nuclei (in myotubes) out of total amount of nuclei) after MP differentiation in 2D. Per cell line, 5 random fields were analyzed. Each dot represents one random field. **(E, F, G)** Gene expression analysis of *DUX4* (**E**), *ZSCAN4* (**F**), and *MYH3* (**G**) from differentiated MPs in 2D myotube cultures

using RT-qPCR. Gene expression is shown as relative expression to the housekeeping gene *GUSB*. Each dot represents one biological replicate and the error bars denote the SD. **(B, D, E, F, G)** Statistical analysis was performed using Student's t-tests. ns = not significant. *p<0.05, **p<0.01, ***p<0.001, ****p<0.0001.

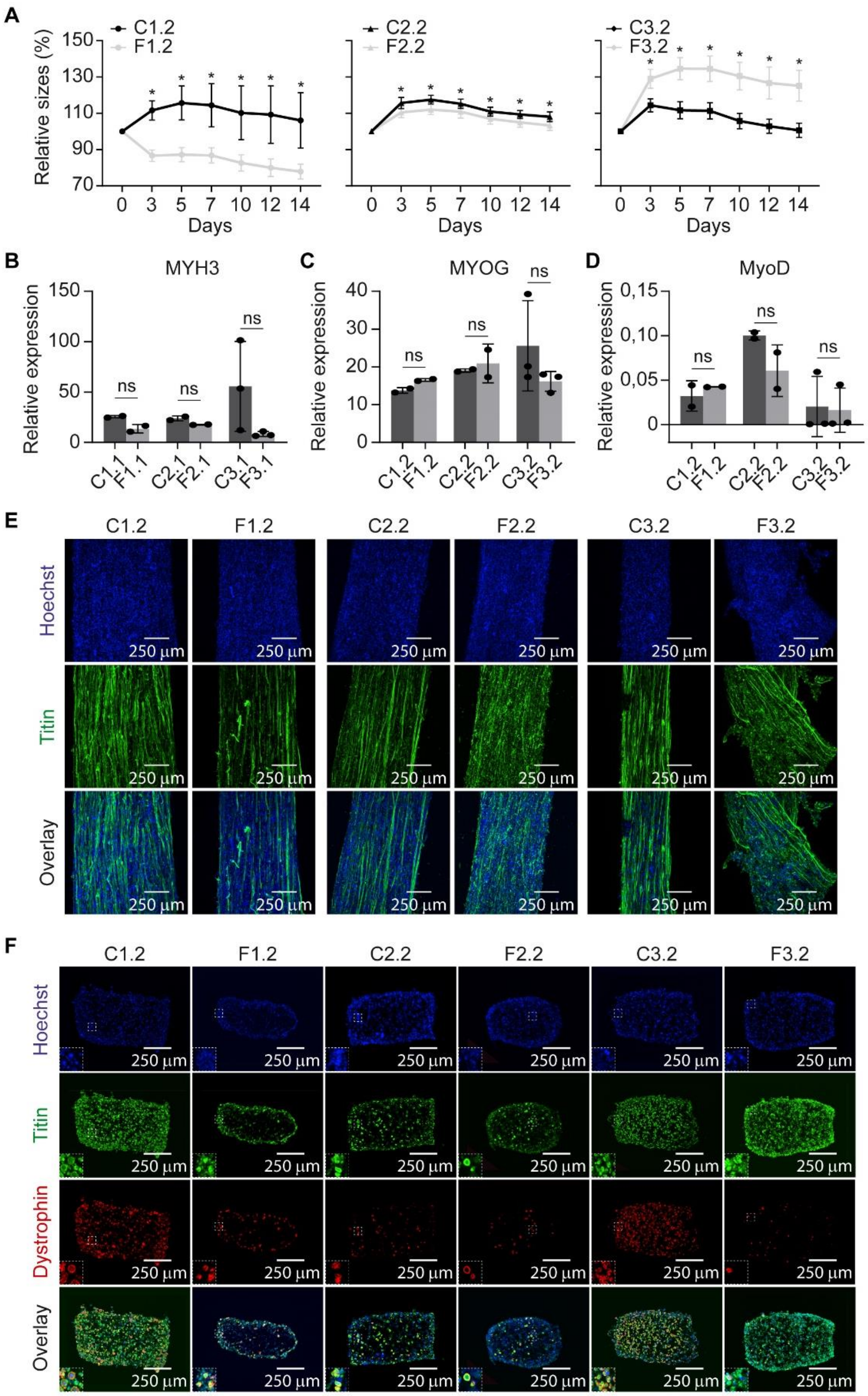

Supplementary Figure 6 Characterization of the second pair of non-affected and affected myogenic progenitors from mosaic FSHD1 patients in 3D-TESMs.

(A) Relative width sizes of non-affected (C) and affected (F) 3D-TESMs from patient 1 clone 2 (C1.2 and F1.2), patient 2 clone 2 (C2.2 and F2.2), and patient 3 clone 2 (C3.2 and F3.2) over time. Data is shown as average of 12 3D-TESMs per line, with error bars denoting SD. Statistical analysis was performed using a Student's t-test. Relative width sizes were normalized to day 0 of differentiation. ns = not significant; *p <0,05 **(B, C, D)** Gene expression analyses of *MYH3* (**B**), *MYOG* (**C**), and *MYOD* (**D**) from non-affected and affected 3D-TESMs differentiated for 14 days by RT-qPCR. Gene expression is shown as relative expression to the housekeeping gene *GUSB*. Statistical analysis was performed using Student's t-tests. Each dot represents one biological replicate and the error bars denote the SD. ns = not significant, *p<0.05, ***p<0.001, ****p<0.0001. **(E)** Representative images of whole mount stainings of 3D-TESMs differentiated for 14 days. Immunofluoresence staining was performed with Hoechst (blue) and anti-titin antibody (green). **(F)** Representative images of cross-sections from non-affected and affected 3D-TESMs differentiated for 14 days. Cross-sections were stained with Hoechst (blue), anti-titin (green), and anti-dystrophin (red) antibodies.

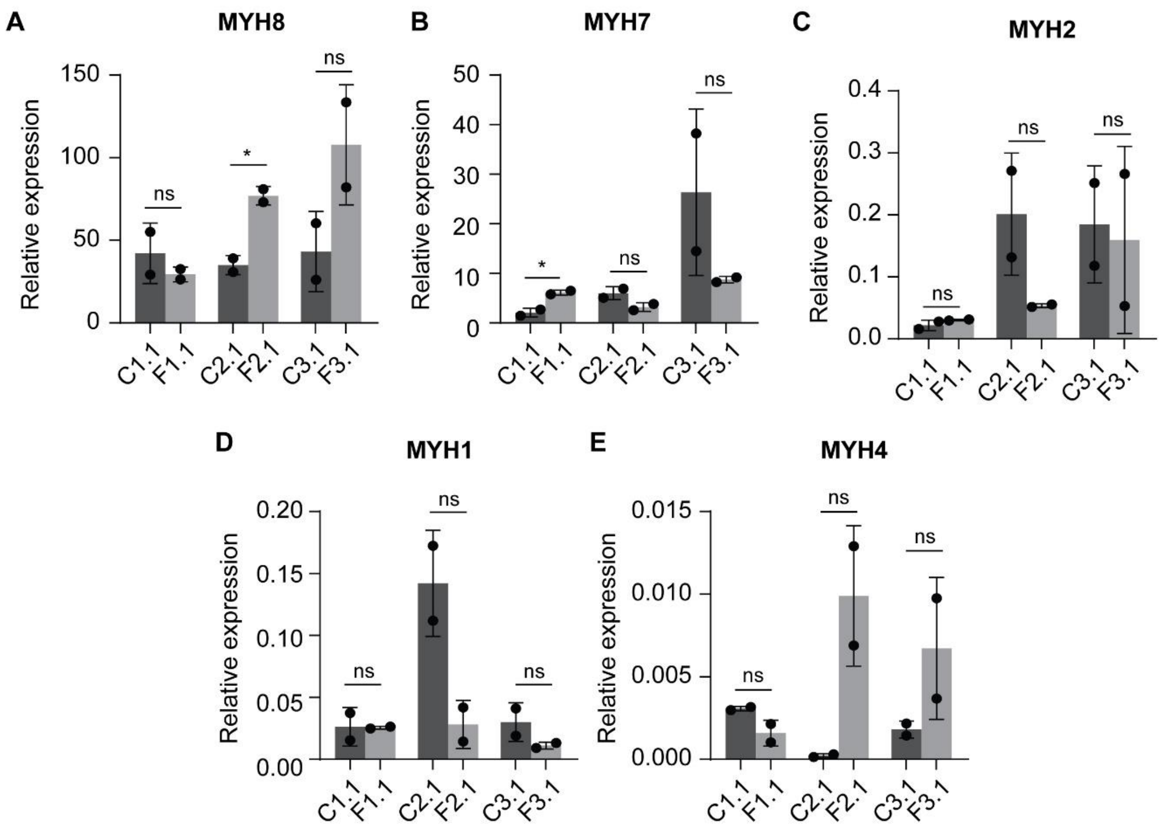

Supplementary Figure 7 Expression of myosin heavy chain isoforms in non-affected and affected 3D-TESMs.

(**A-E**) Gene expression analyses of *MYH8* (**A**), *MYH7* (**B**), *MYH2* (**C**), *MYH1* (**D**) and *MYH4* (**E**) from 3D-TESMs using RT-qPCR. Gene expression is shown as relative expression to housekeeping gene *GUSB*. Each dot represents one biological replicate and the error bars denote the SD. Statistical analysis was performed using Student's t-tests. ns = not significant, *p<0.05.

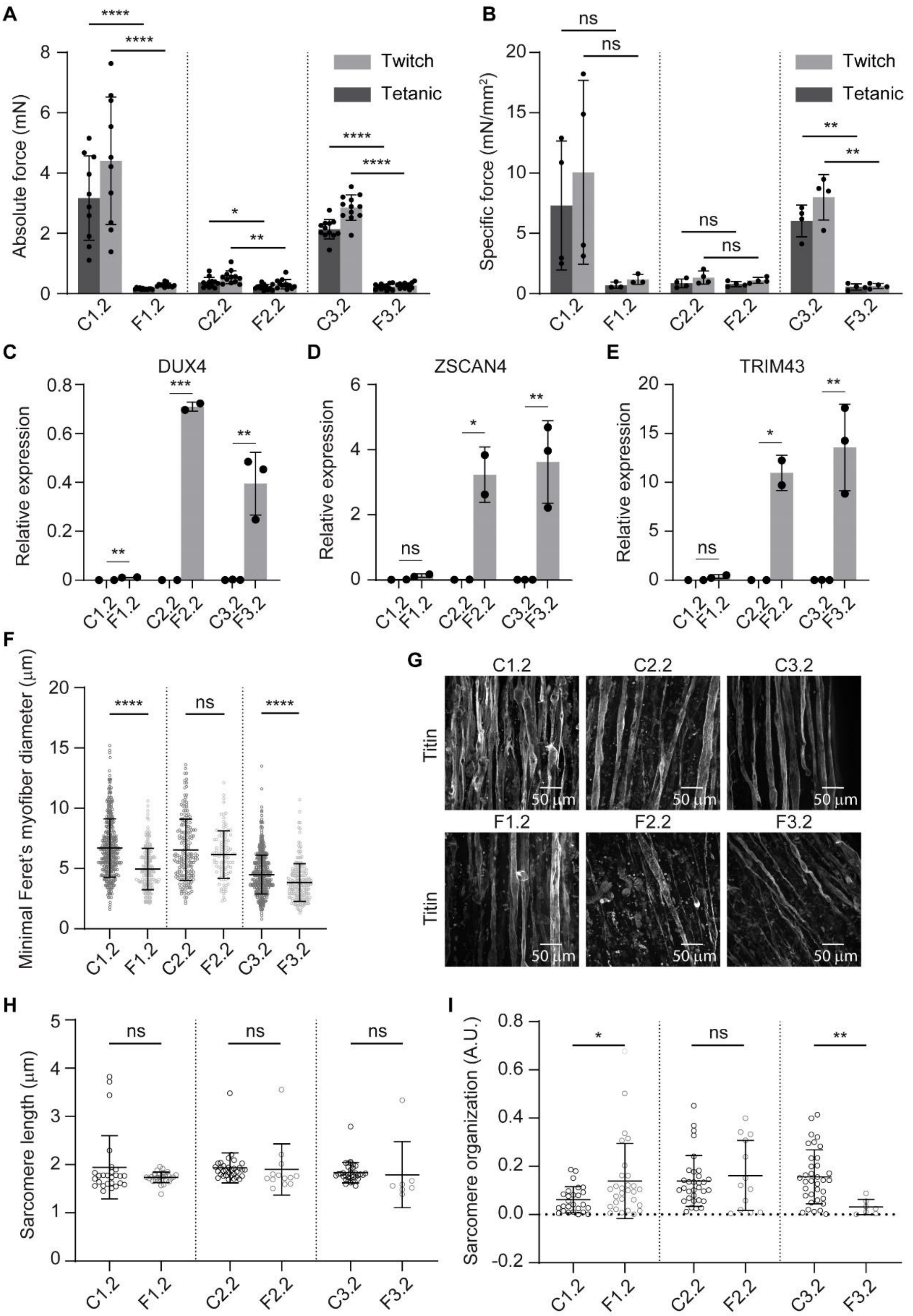

Supplementary Figure 8 Differences between the second pair of non-affected and affected 3D-TESMs from mosaic FSHD1 patients differentiated for 14 days.

(A) Absolute forces of non-affected and affected 3D-TESMs after electrical stimulation at 1 Hz (twitch; grey bars) and 20 Hz (tetanic; dark grey bars). Each dot represents one biological replicate and the error bars denote the SD. **(B)** Specific forces of 3D-TESMs as in A, normalized for their cross-sectional area. Each dot represents one biological replicate and the error bars denote the SD. **(C, D, E)** Gene expression analyses of *DUX4* (**C**), *ZSCAN4* (**D**), and *TRIM43* (**E**) from 3D-TESMs using RT-qPCR. Gene expression is shown as relative expression to housekeeping gene *GUSB*. Each dot represents one biological replicate and the error bars denote the SD. **(F)** Quantification of minimal Feret's myofiber diameter (μm) from myofibers stained for dystrophin in 3D-TESM cross sections. 3D-TESMs were differentiated for 14 days. A minimum of 100 myofibers was analyzed in $n \geq 3$ biological replicates per cell line. Values are shown as mean +/- SD. **(G)** Representative images of whole mount-stained 3D-TESMs at 40x magnification. 3D-TESMs were stained for titin (white). **(H, I)** Quantification of sarcomere length (μm) (**H**) and sarcomere organization score (A.U.) (**I**) using SotaTool software. A minimum of 30 myofibers from one biological replicate was analyzed per condition. **(A, B, C, D, E, F, H, I**) Statistical analysis was performed using Student's t-tests. ns = not significant, $*p<0.05$, $**p<0.01$, $**p<0.01$, $***p<0.001$, $****p<0.0001$.

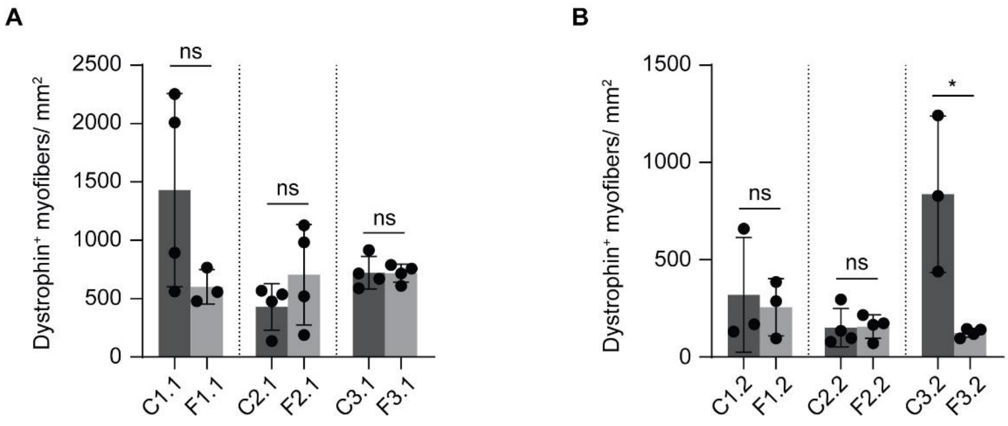

Supplementary Figure 9 (A, B) Quantification of dystrophin-positive myofiber per cross-sectional area from the first pair (**A**) and second pair (**B**) of genetically matched non-affected (C) and affected (F) 3D-TESMs from mosaic FSHD1 patients differentiated for 14 days. Each dot represents one biological replicate and the error bars denote the SD. Statistical analysis was performed using Student's t-tests. ns = not significant, *$p<0.05$.

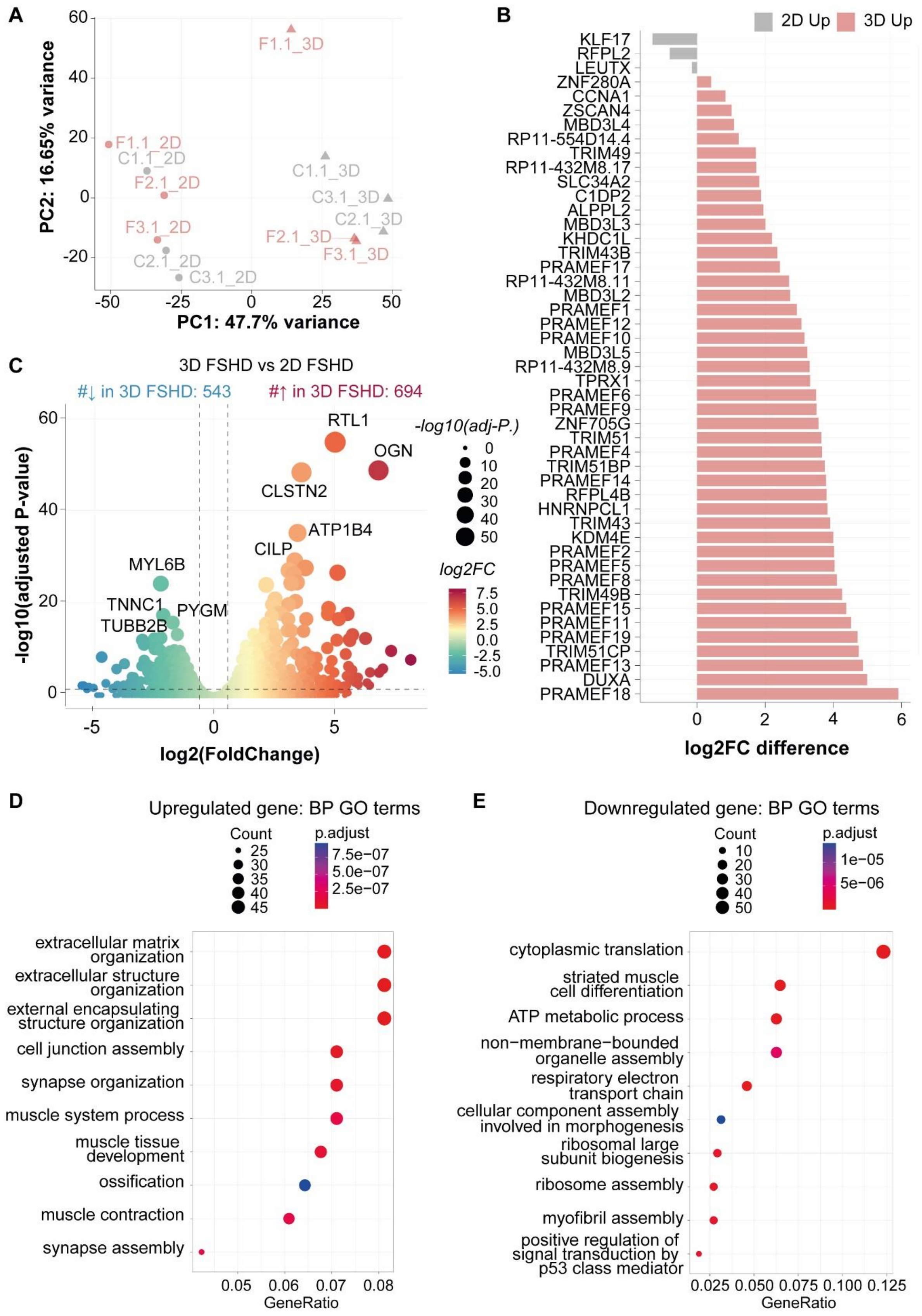

Supplementary Figure 10 RNA sequencing reveals increased DUX4 target genes in affected 3D-TESMs compared to 2D myotubes.

(A) Dot plot showing the result of PCA analysis. Color code represents the condition and shapes of dots represent the culture condition. **(B)** Bar plot showing the log2Foldchange difference of each DUX4 target gene between 2D culture and 3D culture. Color codes represent the direction of log2Foldchange difference. **(C)** Volcano plot displaying the results of differential gene expression analysis between 2D FSHD samples and 3D FSHD samples. Color scales depicts the log2Foldchange of each gene and the size of dots shows the value of –log10(adjusted P-value). The cut-off for the significantly expressed genes is following that |log2(Foldchange)| < log2(1.5) and adjusted P-value < 0.05. **(D, E)** Dot plot illustrating the BP GO terms enriched for upregulated genes and downregulated genes in 3D FSHD samples. Color scales depicts the adjusted P-value. Size of dots shows the number of genes identified for each GO term.

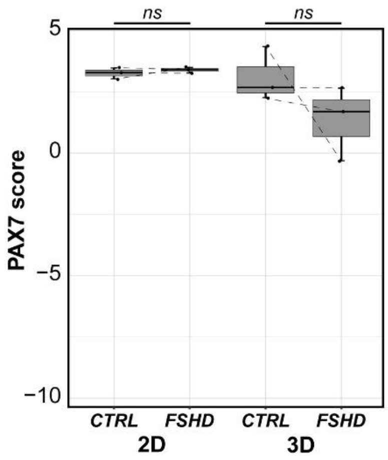

Supplementary Figure 11 PAX7 score analysis in affected and non-affected 2D myotubes and 3D-TESMs. PAX7 scores were calculated on log-transformed and quantile-normalized data, as described by Banerji *et al.*[4] Statistics indicate results of a Student's t-test. ns = not significant, *p<0.05. Dashed lines indicated paired control and FSHD samples, originating from the same mosaic patient biopsy.

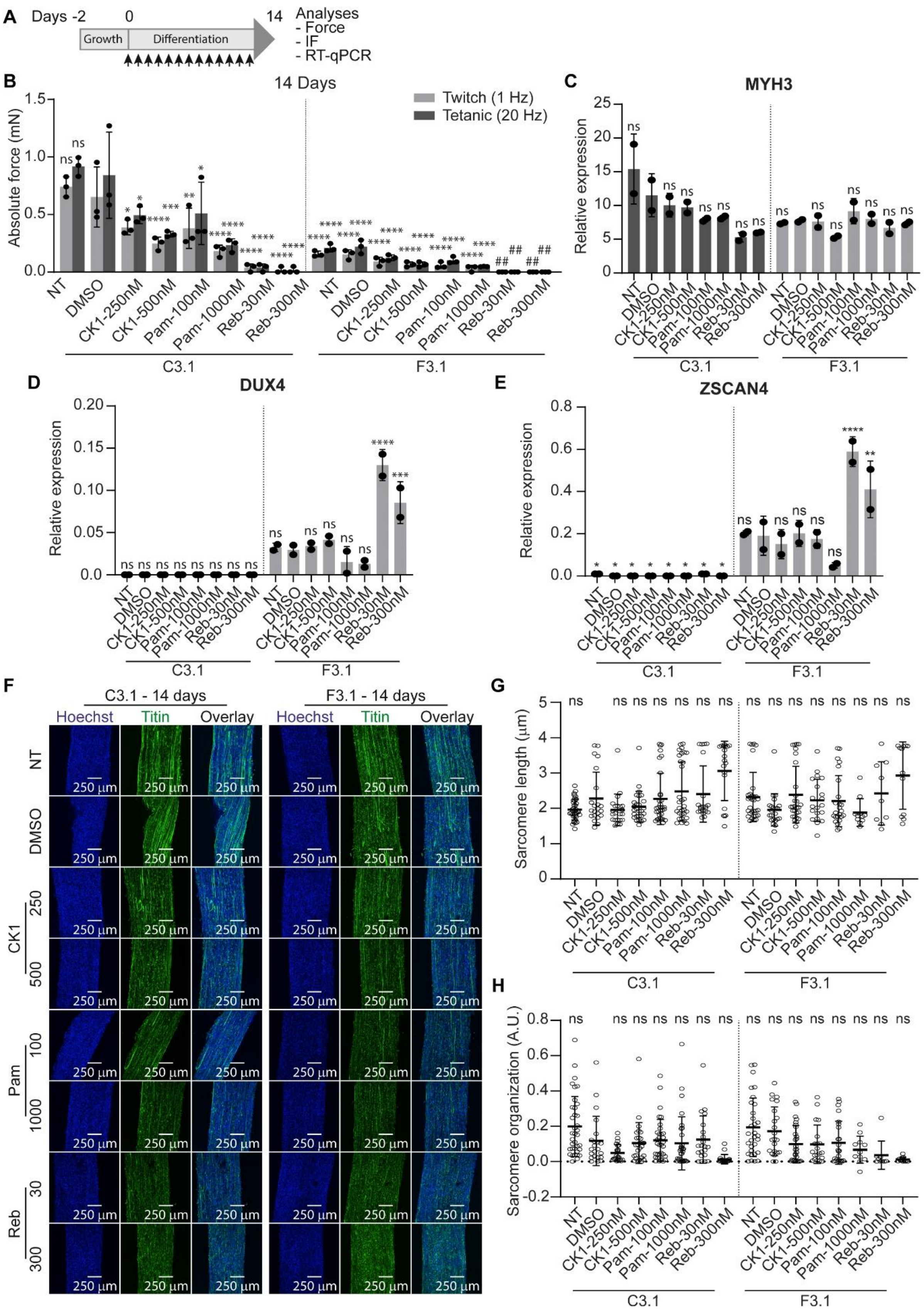

Supplementary Figure 12 Treatment of non-affected and affected myogenic progenitors from mosaic FSHD1 patients in 3D-TESMs with DUX4 inhibitors for 14 days. Non-

affected and affected 3D-TESMs of mosaic FSHD1 patient 3 (C3.1 and F3.1) were non-treated (NT) or treated daily starting at initiation of differentiation for 14 days with DMSO, CK1 inhibitor (final concentration 250 and 500 nM), pamapimod (Pam; final concentrations 100 and 1000 nM), or rebastinib (Reb; 30 and 300 nM).

(A) Timeline of treatment of 3D-TESMs. Cells were grown for two days in proliferation medium, after which medium was changed to 3D differentiation medium supplemented with 10 µM SB431542 and DUX4 inhibitors. Differentiation medium containing DUX4 inhibitors was replaced every day. On day 14 of differentiation, 3D-TESMs were subjected to electrical stimulation for contractile force measurements. Thereafter, 3D-TESMs were either fixed for immunofluorescence staining or harvested for RNA. **(B)** Absolute forces after electrical stimulation at 1 Hz (twitch; grey bars) or 20 Hz (tetanic; dark grey bars). Each dot represents one biological replicate and the error bars denote the SD. **(C, D, E)** Gene expression analyses of *MYH3* (**C**), *DUX4* (**D**), and *ZSCAN4* (**E**) in treated C3.1 and F3.1 3D-TESMs using RT-qPCR. Gene expression is shown as relative expression to the housekeeping gene *GUSB*. Each dot represents one biological replicate and the error bars denote the SD. **(F)** Representative images of whole mount staining of treated 3D-TESMs from C3.1 and F3.1. Immunofluorescence staining was performed with Hoechst (blue) and anti-titin (green). **(G, H)** Quantification of sarcomere length (µm) and sarcomere organization score (A.U.) of single fibers from images shown in panel F using SotaTool software. For each condition, a minimum of 10 fibers was analyzed from one biological replicate. (**B, C, D, E, G, H**) Significance was determined using one-way ANOVA with Bonferroni multiple comparison correction for DMSO-treated non-affected (**B, G, H**) or affected (**C, D, E**) 3D-TESMs. ns = not significant, *p<0.05, **p<0.01, ***p<0.001, ****p<0.0001.

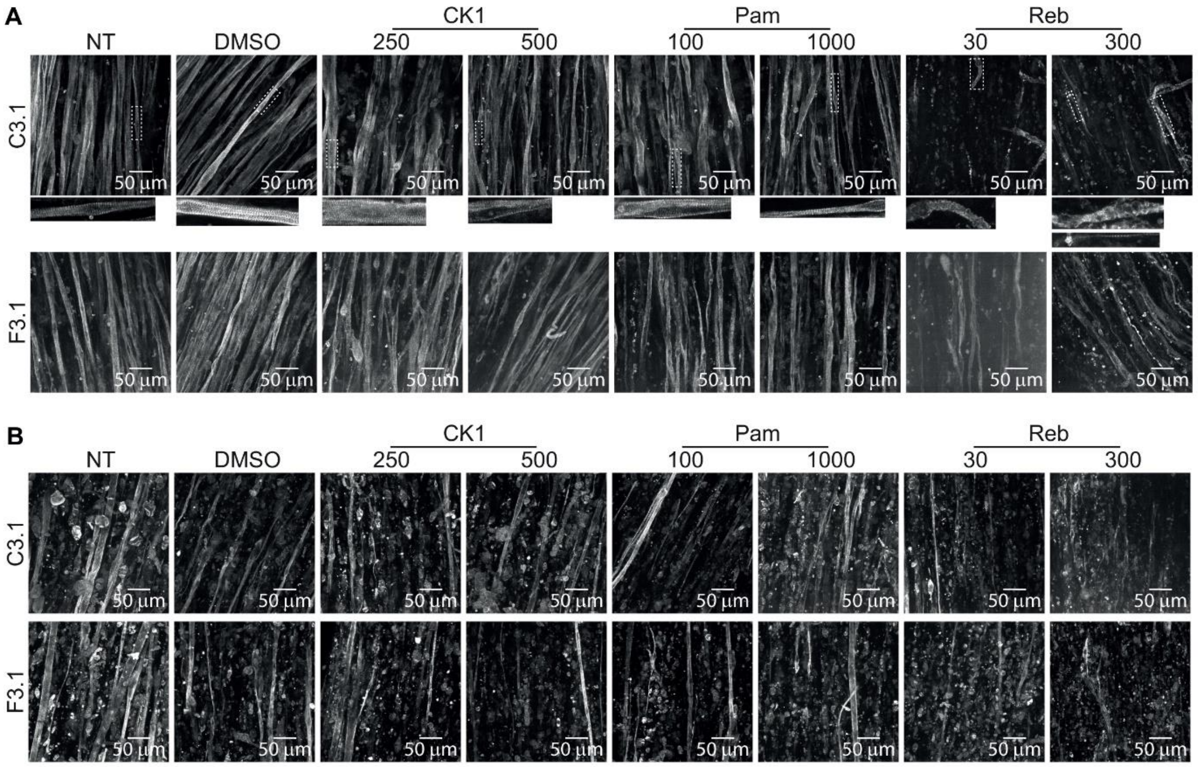

Supplementary Figure 13 Representative images of whole mount titin immunofluorescence staining of 4 days (A) or 14 days (B) treated 3D-TESMs from non-affected and affected MPs of patient 3 (C3.1 and F3.1). 3D-TESMs were treated daily from initiation of differentiation with CK1 inhibitor (final concentration 250 and 500 nM), pamapimod (Pam; final concentrations 100 and 1000 nM), or rebastinib (Reb; 30 and 300 nM).

Supplementary Tables

Supplementary Table 1. Patient information. ACSS = Age Corrected Clinical Severity Score.

	Gender	ACCSS	D4Z4 repeat array (units) allele 1		Allele 1 (Permissive)	D4Z4 repeat array (units) allele 2	Allele 2 (Nonpermissive)
			FSHD1	Control			
Patient 1	Male	38	3	45	4qA161L	38	4qB168
Patient 2	Male	133	2	43	4qA161S	21	4qB163
Patient 3	Male	121	3	19	4qA161S	28	4qB163

Supplementary Table 2. Human induced pluripotent stem cell (hiPSC) information.

	Official name	Non-affected		Affected	
		Clone 1	Clone 2	Clone 1	Clone 2
Patient 1	LUMC0162	iCTRL5	iCTRL2	iFSHD4	iFSHD3
Patient 2	LUMC0163	iCTRL7	iCTRL5	iFSHD4	iFSHD1
Patient 3	LUMC0177	iCTRL2	iCTRL6	iFSHD3	iFSHD1

Supplementary Table 3. List of primer sets for RT-qPCR analysis.

Target	Forward primer sequence (5' à 3')	Reverse primer sequence (5' à 3')
DUX4	CTTCCGTGAAATTCTGGCTGAATG	TTTTTTTTTTTTTTTTTCTATAGGATCCACAGG
ZSCAN4	TGGAAATCAAGTGGCAAAAA	CTGCATGTGGACGTGGAC
TRIM43	ACCCATCACTGGACTGGTGT	CACATCCTCAAAGAGCCTGA
MYH3	CTTGTGGGCGGAGGTCTG	AGCAGCTATGCCGAACACTT
MyoD	TACGAAGGCGCCTACTACAAC	AGGCAGTCTAGGCTCGACAC
MyoG	CGAATGCAGCTCTCACAGCG	CCGTGAGCAGATGATCCCC
GUSB	CTCATTTGGAATTTTGCCGATT	CCGAGTGAAGATCCCCTTTTTA

Supplementary Table 4. List of primary and secondary antibodies used.

Primary antibodies

Target	*Host*	*Dilution*	*Company*	*Cat. number*	*IF,IHC*
Titin	Mouse IgM	1:50	DSHB	9D10-S	IF, IHC
Dystrophin	Rabbit	1:100	Abcam	Ab15277	IHC
Myosin	Mouse IgG2b	1:50	DSHB	MF20	IF
NCAM	Mouse IgG1	1:200	Cell Signalling	3576	IF
NANOG	Mouse IgG1	1:150	Santa Cruz	Sc-293121	IF
OCT4	Mouse IgG2b	1:200	Stemcell Technologies	60093	IF
SSEA4	Mouse IgG3	1:30	Biolegend	330402	IF
Vimentin	Mouse IgG1	1:50	Sigma-Aldrich	V6630	IF
PAX6	Rabbit	1:200	Cell Signaling	60433	IF
FOXA2	Rabbit	1:100	Millipore	07-633	IF

Secondary antibodies

Target	*Dilution*	*Company*	*Cat. number*	*IF,IHC*
Goat anti-mouse IgM Alexa Fluor 488	1:500	Invitrogen	A-21042	IF, IHC
Donkey anti-rabbit Alexa Fluor 594	1:500	Invitrogen	A-21207	IF, IHC
Goat anti-mouse IgG1b Alexa fluor 488	1:500	Invitrogen	A-21121	IF
Goat anti-mouse IgG2b Alexa fluor 594	1:500	Invitrogen	A-21145	IF
Hoechst 34580	1:10,000	ThermoFisher	H21486	IF, IHC

Supplementary References

1. van der Wal E, den Hamer B, van der Vliet PJ, et al. Generation of genetically matched hiPSC lines from two mosaic facioscapulohumeral dystrophy type 1 patients. Stem Cell Res. Oct 2019;40:101560. doi:10.1016/j.scr.2019.101560

2. Lemmers RJ. Analyzing Copy Number Variation Using Pulsed-Field Gel Electrophoresis: Providing a Genetic Diagnosis for FSHD1. Methods Mol Biol. 2017;1492:107-125. doi:10.1007/978-1-4939-6442-0_7

3. van der Wal E, Herrero-Hernandez P, Wan R, et al. Large-Scale Expansion of Human iPSC-Derived Skeletal Muscle Cells for Disease Modeling and Cell-Based Therapeutic Strategies. Stem Cell Reports. Jun 5 2018;10(6):1975-1990. doi:10.1016/j.stemcr.2018.04.002

4. Banerji CRS, Panamarova M, Hebaishi H, et al. PAX7 target genes are globally repressed in facioscapulohumeral muscular dystrophy skeletal muscle. Nat Commun. Dec 18 2017;8(1):2152. doi:10.1038/s41467-017-01200-4
